# Supplementary material for: Study design features increase replicability in brain-wide association studies
Source: Nature. 2024 Nov 27;636(8043):719–27. doi: 10.1038/s41586-024-08260-9 (PMC11655360; doi:10.1038/s41586-024-08260-9)
Supplement: Supplementary file 1 — Supplementary Information [file 41586_2024_8260_MOESM1_ESM.pdf]

nature portfolio

https://doi.org/10.1038/s41586-024-08260-9

Supplementary information

Study design features increase replicability in brain-wide association studies

In the format provided by the authors and unedited

Supplementary Information for *Study design features increase replicability in brain-wide association studies*

Kaidi Kang[1,*], Jakob Seidlitz[2,3,4], Richard A.I. Bethlehem[5], Jiangmei Xiong[1], Megan T. Jones[1], Kahini Mehta[3,4,6], Arielle S. Keller[7,8], Ran Tao[1,9], Anita Randolph[10,11], Bart Larsen[10,11], Brenden Tervo-Clemmens[11,12], Eric Feczko[10,11], Oscar Miranda Dominguez[10,11], Steve M. Nelson[10,11], Lifespan Brain Chart Consortium, 3R-BRAIN, AIBL, Alzheimer's Disease Neuroimaging Initiative, Alzheimer's Disease Repository Without Borders Investigators, CALM Team, CCNP, COBRE, cVEDA, Harvard Aging Brain Study, IMAGEN, POND, The PREVENT-AD Research Group, Jonathan Schildcrout[1], Damien A. Fair[10,11,13], Theodore D. Satterthwaite[3,4,6], Aaron Alexander-Bloch[2,3,4,†], and Simon Vandekar[1,†,*]

[1]Department of Biostatistics, Vanderbilt University Medical Center
[2]Department of Child and Adolescent Psychiatry and Behavioral Sciences, The Children's Hospital of Philadelphia
[3]Department of Psychiatry, University of Pennsylvania
[4]Lifespan Brain Institute of The Children's Hospital of Philadelphia and Penn Medicine
[5]Department of Psychology, University of Cambridge
[6]Penn Lifespan Informatics and Neuroimaging Center (PennLINC), Perelman School of Medicine, University of Pennsylvania
[7]Department of Psychological Sciences, University of Connecticut
[8]Institute for the Brain and Cognitive Sciences, University of Connecticut
[9]Vanderbilt Genetics Institute, Vanderbilt University Medical Center
[10]Department of Pediatrics, University of Minnesota Medical School
[11]Masonic Institute for the Developing Brain, University of Minnesota
[12]Department of Department of Psychiatry & Behavioral Sciences, University of Minnesota Medical School
[13]Institute of Child Development, University of Minnesota

† These authors contributed equally
* Corresponding authors:
Kaidi Kang (kaidi.kang@vanderbilt.edu)
Simon Vandekar (simon.vandekar@vumc.org)

Contents

1 Supplementary Figures **4**
Figure S1 4
Figure S2 5
Figure S3 6
Figure S4 7

2 Supplementary Tables **8**
Table S1 8
Table S2 9
Table S3 10
Table S4 10
Table S5 10
Table S6 10
Table S7 11
Table S8 11
Table S9 11
Table S10 11
Table S11 12
Table S12 13
Table S13 14

3 Robust Effect Size Index (RESI) in Longitudinal Analysis **15**
3.1 RESI for generalized estimating equations 15
3.1.1 Longitudinal RESI (L-RESI) 16
3.1.2 "Cross-sectional" RESI (CS-RESI) in longitudinal analysis 17
3.1.3 Confidence interval construction for CS-RESI 18
3.2 Simulation Studies 19
3.2.1 Simulation setup 19
3.2.2 Simulation results 19
Figure S5 21
Figure S6 22
Figure S7 23
Figure S8 24
Figure S9 25
3.3 Comparison of L-RESI and CS-RESI quantifies the benefit of longitudinal design 26
Table S14 26
Table S15 26
Table S16 26
Table S17 27
Figure S10 28
Figure S11 29
Figure S12 30
Figure S13 31

4 Modified Sampling Design Considerations **31**
4.1 Sampling estimates for targeted sampling 31
Table S16 32
4.2 Implications for multivariate BWAS 32

5 Longitudinal Design and Analysis Considerations **32**
Figure S14 . . . 33
5.1 Equal between- and within-subject effects . . . 35
5.2 Modeling different between- and within-subject effects . . . 37
5.2.1 Interpretation of between- and within-subject effects . . . 38
5.3 Modeling the averaged parameter . . . 39
Table S19 . . . 40
5.4 Derivation of effect size under exchangeable design . . . 40

6 Sample Size Code for Modified Sampling **41**

7 Consortium Member Information **43**

1 Supplementary Figures

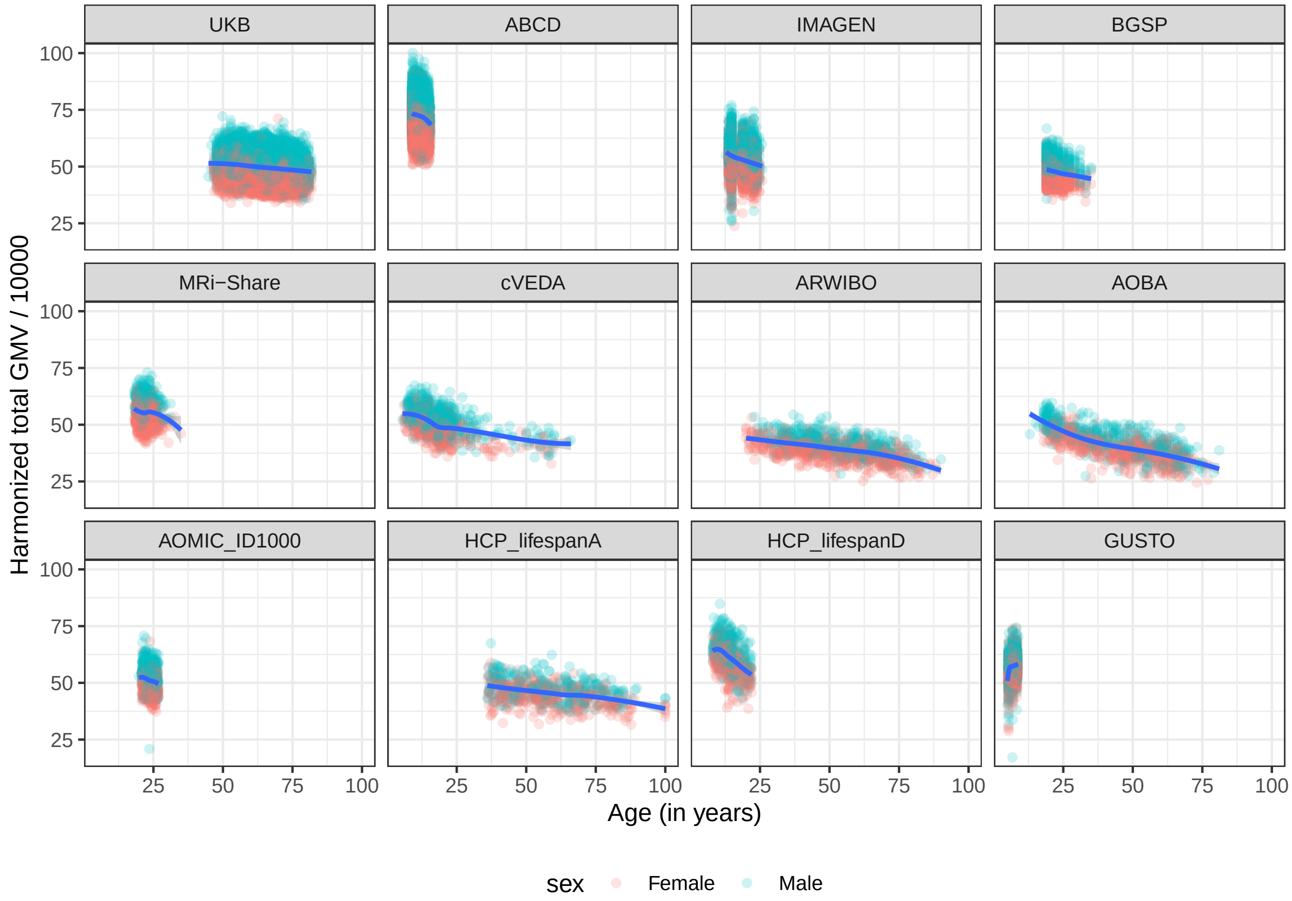

Figure S1: Scatterplots of the harmonized total gray matter volume (GMV) measures (i.e., total GMV after removing site effects using ComBat or longCombat) and age (in years) in the 12 largest studies among the 63 studies. The blue curves are the locally estimated scatterplot smoothing (LOESS) curves. The shaded areas are the 95% confidence bands. MRI datasets containing 77,695 scans from 63 studies with different age sample distributions were used. Nonlinear models are used to fit total GMV, white matter volume (WMV), subcortical gray matter volume (sGMV), and mean cortical thickness (CT) as a function of age (in years) and sex in each study.

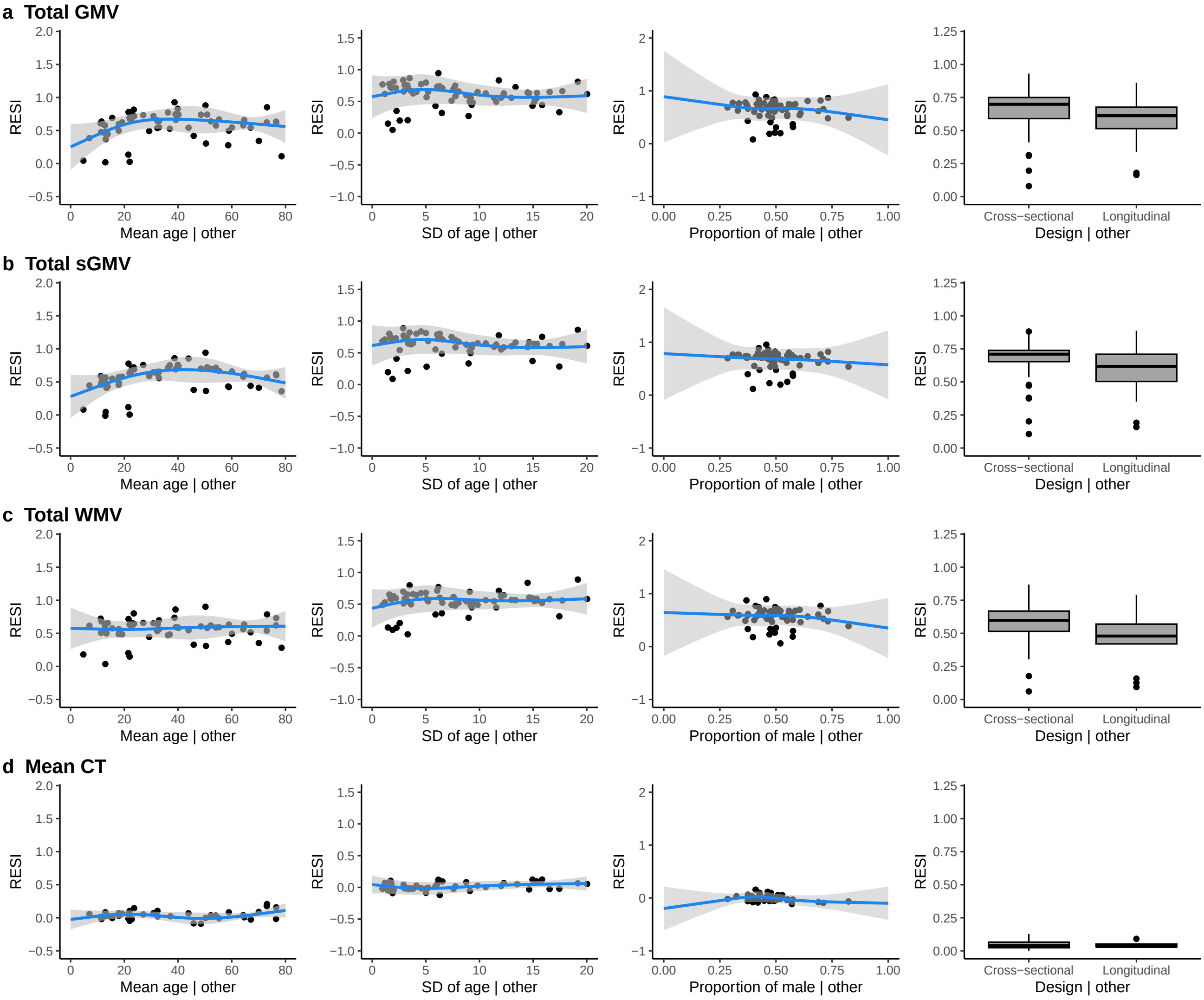

Figure S2: Meta-analysis results regarding the effects of study features on the associations between sex and different brain measures. (a-d) Partial regression plots of the meta-analyses of standardized effect sizes (i.e., RESI) for the associations between sex and global brain measures (a) total gray matter volume (GMV), (b) total subcortical gray matter volume (sGMV), (c) total white matter volume (WMV), and (d) mean cortical thickness (CT) ("| Other" means after fixing the other features at constant levels: design = cross-sectional, mean age = 45 years, sample age standard deviation (SD) = 7 and/or proportion of males = 0.5). The blue curves are the expected standardized effect size for sex. The gray areas are the 95% confidence bands.

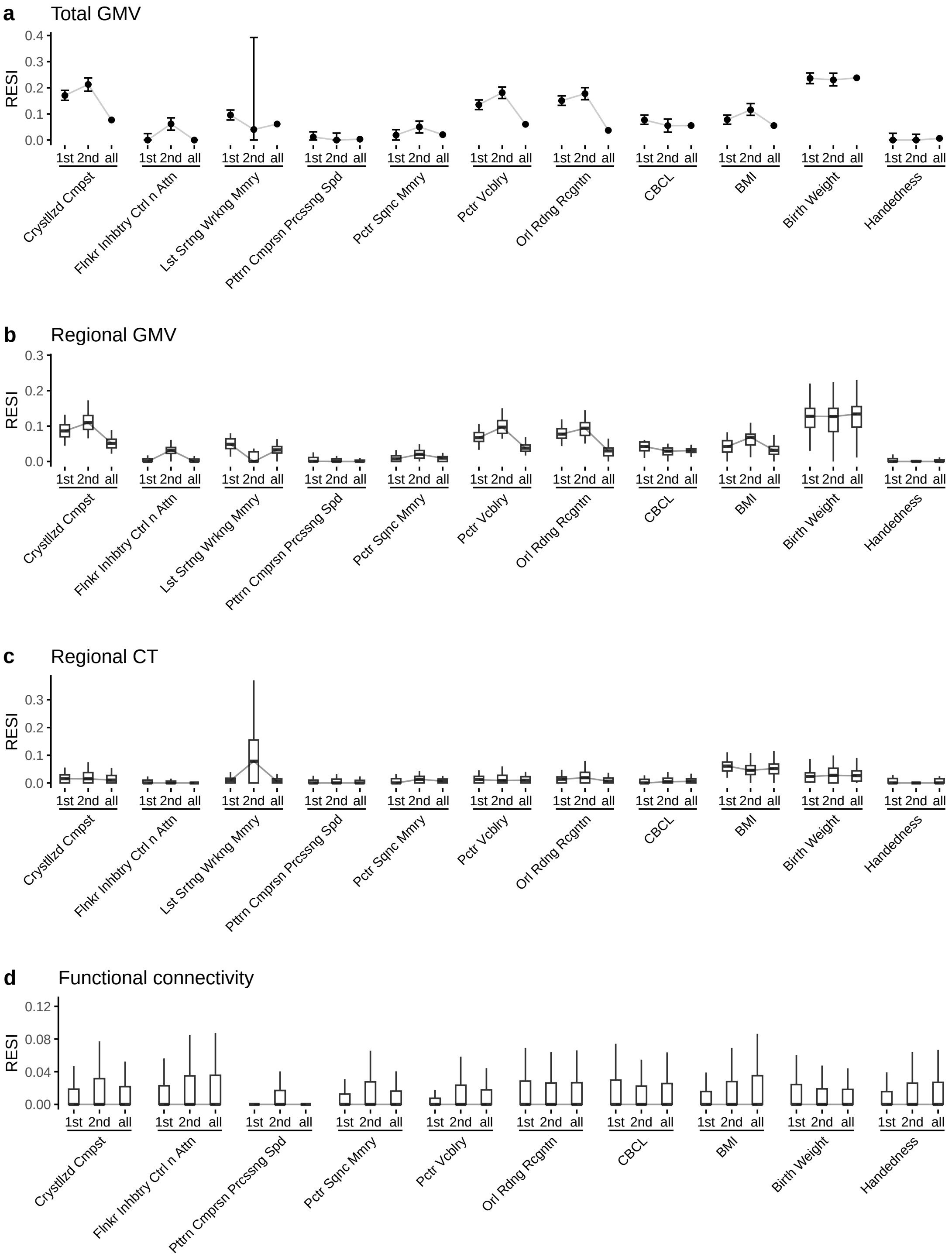

Figure S3: Replication of Extended Data Figure. 4 using linear mixed models (LMMs) with individual-specific random intercepts. Boxplots show the distributions of the standardized effect sizes (ESs) across regions. The estimates for the RESI from cross-sectional analyses that only use the baseline measures or the first follow-up measures after baseline are indicated by "1st"s or "2nd"s on the x-axes, respectively; the estimates for RESI from longitudinal analyses that use the full longitudinal data are indicated by "all"s on the x-axes. The results are similar using generalized estimating equations (GEEs) and LMMs and show that longitudinal study designs can reduce standardized ESs for most structural brain associations. Only the point estimates of RESI are obtained as the bootstrap confidence intervals (CIs) in LMMs have not been evaluated.

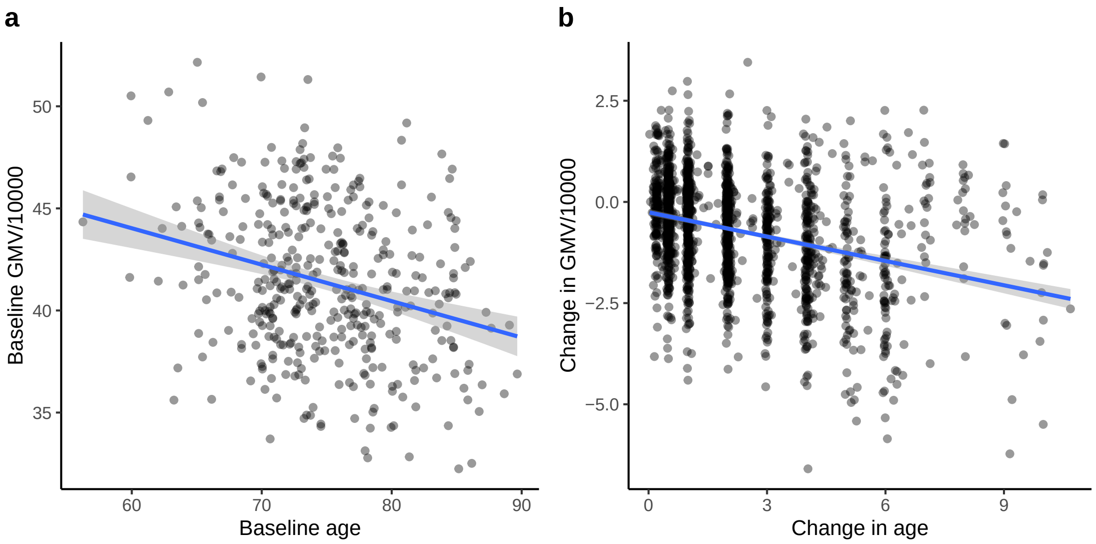

Figure S4: Visualization of the strengths of crude between- and within-participant associations between age and total gray matter volume (GMV) in ADNI. There are strong associations (a) between baseline total GMV and baseline age and (b) between change in total GMV and change in age since baseline, indicating there exist strong between- and within-subject effects of age on total GMV in ADNI study. The blue lines are the simple linear regression lines and the gray areas are the 95% confidence bands.

2 Supplementary Tables

Table S1: The descriptive summary of the 63 neuroimaging datasets used in the meta-analyses for the total gray matter volume (GMV), subcortical gray matter volume (sGMV), and white matter volume (WMV).

Study	N	Total number of observations	Design	Mean age	SD of age	Skewness of age	Proportion of males
UKB	29031	29031	Cross-sectional	64.64	7.58	-3.96	0.50
ABCD	8874	17210	Longitudinal	11.18	1.60	1.35	0.50
IMAGEN	2107	4446	Longitudinal	17.98	3.35	2.34	0.48
BGSP	1570	1570	Cross-sectional	21.56	2.89	3.40	0.42
MRi-Share	1341	1341	Cross-sectional	22.01	2.21	2.31	0.31
cVEDA	1193	1193	Cross-sectional	19.19	9.82	12.78	0.58
ARWIBO	1077	1077	Cross-sectional	52.19	15.33	4.37	0.38
AOBA	964	964	Cross-sectional	43.90	15.83	-3.36	0.47
AOMIC_ID1000	925	925	Cross-sectional	23.63	1.71	0.76	0.48
HCP_lifespanA	689	689	Cross-sectional	58.92	14.94	10.75	0.43
HCP_lifespanD	655	655	Cross-sectional	13.79	3.81	3.00	0.49
GUSTO	642	1230	Longitudinal	6.98	1.18	-0.73	0.51
OASIS3	600	600	Cross-sectional	67.01	9.18	-4.27	0.40
ICBM	588	588	Cross-sectional	30.84	12.27	14.27	0.52
SLIM	562	981	Longitudinal	21.48	1.46	1.44	0.47
abide1	560	560	Cross-sectional	17.88	7.76	8.90	0.82
IXI	538	538	Cross-sectional	48.52	16.54	4.59	0.42
ADHD200	481	481	Cross-sectional	12.92	3.31	3.24	0.52
SALD	473	473	Cross-sectional	45.82	17.44	4.61	0.37
AIBL	470	822	Longitudinal	73.02	6.54	4.59	0.46
devCCNP	423	423	Cross-sectional	12.94	2.93	2.08	0.47
NIHPD	423	882	Longitudinal	12.94	4.12	2.52	0.47
ADNI	417	2232	Longitudinal	76.57	6.06	1.48	0.51
NHGRI	410	410	Cross-sectional	22.71	14.63	16.09	0.57
abide2	391	391	Cross-sectional	17.94	10.58	12.82	0.71
PREVENTAD	383	1678	Longitudinal	64.28	5.21	5.09	0.28
3R-BRAIN	330	330	Cross-sectional	27.05	4.55	3.60	0.56
BHRCS	319	642	Longitudinal	13.35	3.61	2.63	0.55
DLBS	313	313	Cross-sectional	55.26	20.03	-7.84	0.37
Oslo	280	280	Cross-sectional	50.83	17.72	-7.31	0.41
HABS	277	670	Longitudinal	76.48	6.29	4.64	0.42
FemaleASD	229	229	Cross-sectional	12.75	3.03	1.02	0.48
AOMIC_PIOP2	224	224	Cross-sectional	21.97	1.79	0.77	0.43
IMAP	214	418	Longitudinal	50.21	19.16	-7.41	0.46
CUBA	201	201	Cross-sectional	32.26	9.36	8.50	0.73
SWU_MDD	200	200	Cross-sectional	40.17	15.35	6.00	0.34
WAYNE	199	291	Longitudinal	54.10	15.11	-12.16	0.33
BSNIP	197	197	Cross-sectional	39.18	12.98	-1.84	0.44
Narratives	181	181	Cross-sectional	22.75	5.00	6.68	0.45
EDSD	162	162	Cross-sectional	70.05	5.90	-2.88	0.48
Pixar	155	155	Cross-sectional	11.33	8.05	9.28	0.46
Psyscan_Dublin	154	154	Cross-sectional	32.93	11.36	11.36	0.47
Maastricht	143	143	Cross-sectional	29.26	9.27	7.63	0.48
LA5c	125	125	Cross-sectional	32.32	8.77	7.45	0.53
SCZIowa	106	223	Longitudinal	32.91	9.09	7.54	0.61
PPMI	105	105	Cross-sectional	60.06	11.56	-9.29	0.61
POND	97	109	Longitudinal	13.05	5.07	3.43	0.55
MCIC	90	90	Cross-sectional	32.41	12.01	8.87	0.69
COBRE	86	86	Cross-sectional	38.69	11.80	6.89	0.73
UCSD	81	87	Longitudinal	4.74	2.27	3.85	0.57
CALM	77	77	Cross-sectional	11.51	1.99	1.13	0.49
AOMIC_PIOP1	73	73	Cross-sectional	21.97	1.90	1.04	0.40
LATAM	73	73	Cross-sectional	23.52	3.49	2.51	0.70
CAMFT	68	68	Cross-sectional	15.50	0.96	-0.67	0.49
Cornell_C1	66	66	Cross-sectional	73.12	6.17	-2.77	0.41
BIODEP	55	55	Cross-sectional	36.21	7.74	5.86	0.36
VITA	54	99	Longitudinal	78.53	2.57	2.45	0.49
OpenPain_BN1	39	39	Cross-sectional	39.89	13.36	9.91	0.64
EMBARC	38	38	Cross-sectional	39.04	14.49	10.67	0.37
OpenPain_RS	33	33	Cross-sectional	50.38	8.98	-8.94	0.58
TOPSY	29	29	Cross-sectional	22.34	3.28	0.71	0.59
OpenPain_PLOA	20	20	Cross-sectional	58.68	6.49	6.80	0.50
OpenPain_SAB	20	20	Cross-sectional	36.91	7.40	5.91	0.55

Table S2: Descriptive summary table for the 43 studies used in the meta-analyses for mean cortical thickness (CT), regional gray matter volume (GMV), and regional CT.

Study	N	Total number of observations	Design	Mean age	SD of age	Skewness of age	Proportion of males
UKB	29030	29030	Cross-sectional	64.64	7.58	-3.96	0.50
ABCD	8874	17210	Longitudinal	11.18	1.60	1.35	0.50
IMAGEN	2105	4437	Longitudinal	17.99	3.35	2.33	0.48
BGSP	1569	1569	Cross-sectional	21.56	2.89	3.40	0.42
cVEDA	1193	1193	Cross-sectional	19.19	9.82	12.78	0.58
ARWIBO	1077	1077	Cross-sectional	52.19	15.33	4.37	0.38
AOBA	964	964	Cross-sectional	43.90	15.83	-3.36	0.47
AOMIC_ID1000	924	924	Cross-sectional	23.63	1.71	0.76	0.48
HCP_lifespanA	689	689	Cross-sectional	58.92	14.94	10.75	0.43
HCP_lifespanD	655	655	Cross-sectional	13.79	3.81	3.00	0.49
GUSTO	642	1228	Longitudinal	6.98	1.18	-0.73	0.51
OASIS3	598	598	Cross-sectional	67.08	9.11	-3.94	0.40
ICBM	588	588	Cross-sectional	30.84	12.27	14.27	0.52
SLIM	562	981	Longitudinal	21.48	1.46	1.44	0.47
abide1	560	560	Cross-sectional	17.88	7.76	8.90	0.82
IXI	538	538	Cross-sectional	48.52	16.54	4.59	0.42
ADHD200	481	481	Cross-sectional	12.92	3.31	3.24	0.52
SALD	473	473	Cross-sectional	45.82	17.44	4.61	0.37
AIBL	469	821	Longitudinal	73.02	6.54	4.59	0.46
devCCNP	422	422	Cross-sectional	12.93	2.93	2.09	0.47
NIHPD	422	881	Longitudinal	12.94	4.12	2.51	0.47
ADNI	417	2232	Longitudinal	76.57	6.06	1.48	0.51
NHGRI	410	410	Cross-sectional	22.71	14.63	16.09	0.57
abide2	391	391	Cross-sectional	17.94	10.58	12.82	0.71
PREVENTAD	383	1677	Longitudinal	64.28	5.21	5.09	0.28
BHRCS	319	638	Longitudinal	13.37	3.61	2.62	0.55
DLBS	313	313	Cross-sectional	55.26	20.03	-7.84	0.37
3R-BRAIN	291	291	Cross-sectional	27.05	4.58	3.66	0.55
HABS	277	670	Longitudinal	76.48	6.29	4.64	0.42
FemaleASD	229	229	Cross-sectional	12.75	3.03	1.02	0.48
AOMIC_PIOP2	224	224	Cross-sectional	21.97	1.79	0.77	0.43
IMAP	214	418	Longitudinal	50.21	19.16	-7.41	0.46
WAYNE	197	287	Longitudinal	54.02	15.20	-12.10	0.32
Narratives	181	181	Cross-sectional	22.75	5.00	6.68	0.45
EDSD	162	162	Cross-sectional	70.05	5.90	-2.88	0.48
LA5c	125	125	Cross-sectional	32.32	8.77	7.45	0.53
BSNIP	99	99	Cross-sectional	37.16	13.51	8.40	0.39
POND	97	109	Longitudinal	13.05	5.07	3.43	0.55
MCIC	90	90	Cross-sectional	32.41	12.01	8.87	0.69
CALM	77	77	Cross-sectional	11.51	1.99	1.13	0.49
AOMIC_PIOP1	73	73	Cross-sectional	21.97	1.90	1.04	0.40
CAMFT	68	68	Cross-sectional	15.50	0.96	-0.67	0.49
Cornell_C1	66	66	Cross-sectional	73.12	6.17	-2.77	0.41

Table S3: Meta-analysis results for the RESI for age on total gray matter volume (GMV). The residual degrees of freedom are 52. The regression model was weighted by the inverse standard errors of the RESIs.

	Estimate	S.E.	F	d.f.	p-value	Meta-RESI	95% CI
Design: longitudinal	0.31	0.10	9.80	1	<0.01	0.37	(0.10, 0.70)
Mean age			5.62	3	<0.01	0.46	(0.09, 0.97)
SD of age			18.90	3	<0.01	0.90	(0.50, 1.67)
Skewness of age			2.34	3	0.08	0.24	(0.00, 0.72)

*S.E.: robust (sandwich) standard error; F: F statistic; d.f.: the first degree(s) of freedom for the F statistics; RESI: robust effect size index; Meta-RESI: the RESI used to quantify the effects from the meta-analysis model; CI: confidence interval; SD: standard deviation.

Table S4: Meta-analysis results for the RESI for age on total subcortical gray matter volume (sGMV). The residual degrees of freedom are 52. The regression model is weighted by the inverse standard errors of the RESIs.

	Estimate	S.E.	F	d.f.	p-value	Meta-RESI	95% CI
Design: longitudinal	0.23	0.11	4.92	1	0.03	0.24	(0.00, 0.78)
Mean age			1.61	3	0.20	0.16	(0.00, 0.67)
SD of age			6.98	3	<0.01	0.52	(0.00, 1.18)
Skewness of age			1.27	3	0.29	0.10	(0.00, 0.63)

*S.E.: robust (sandwich) standard error; F: F statistic; d.f.: the first degree(s) of freedom for the F statistics; RESI: robust effect size index; Meta-RESI: the RESI used to quantify the effects from the meta-analysis model; CI: confidence interval; SD: standard deviation.

Table S5: Meta-analysis results for the RESI for age on total white matter volume (WMV). The residual degrees of freedom are 52. The regression model is weighted by the inverse standard errors of the RESIs.

	Estimate	S.E.	F	d.f.	p-value	Meta-RESI	95% CI
Design: longitudinal	0.42	0.13	11.11	1	<0.01	0.39	(0.21, 0.88)
Mean age			7.50	3	<0.01	0.54	(0.33, 1.39)
SD of age			1.19	3	0.32	0.08	(0.00, 0.75)
Skewness of age			0.30	3	0.83	0.00	(0.00, 0.50)

*S.E.: robust (sandwich) standard error; F: F statistic; d.f.: the first degree(s) of freedom for the F statistics; RESI: robust effect size index; Meta-RESI: the RESI used to quantify the effects from the meta-analysis model; CI: confidence interval; SD: standard deviation.

Table S6: Meta-analysis results for the RESI for age on mean cortical thickness (CT). The residual degrees of freedom are 32. The regression model is weighted by the inverse standard errors of the RESIs.

	Estimate	S.E.	F	d.f.	p-value	Meta-RESI	95% CI
Design: longitudinal	0.25	0.19	1.79	1	0.19	0.13	(0.00, 0.82)
Mean age			2.30	3	0.10	0.28	(0.00, 1.26)
SD of age			4.27	3	0.01	0.46	(0.10, 1.55)
Skewness of age			0.15	3	0.93	0.00	(0.00, 0.67)

*S.E.: robust (sandwich) standard error; F: F statistic; d.f.: the first degree(s) of freedom for the F statistics; RESI: robust effect size index; Meta-RESI: the RESI used to quantify the effects from the meta-analysis model; CI: confidence interval; SD: standard deviation.

Table S7: Meta-analysis results for the RESI for sex on total gray matter volume (GMV). The residual degrees of freedom are 52. The regression model is weighted by the inverse standard errors of the RESIs.

	Estimate	S.E.	F	d.f.	p-value	Meta-RESI	95% CI
Design: longitudinal	-0.03	0.11	0.06	1	0.80	0.00	(0.00, 0.31)
Mean age			0.45	3	0.72	0.00	(0.00, 0.71)
SD of age			0.40	3	0.75	0.00	(0.00, 0.50)
Proportion of male			0.18	3	0.91	0.00	(0.00, 0.49)

*S.E.: robust (sandwich) standard error; F: F statistic; d.f.: the first degree(s) of freedom for the F statistics; RESI: robust effect size index; Meta-RESI: the RESI used to quantify the effects from the meta-analysis model; CI: confidence interval; SD: standard deviation.

Table S8: The meta-analysis results for the RESI for sex on total subcortical gray matter volume (sGMV). The residual degrees of freedom are 52. The regression model is weighted by the inverse standard errors of the RESIs.

	Estimate	S.E.	F	d.f.	p-value	Meta-RESI	95% CI
Design: longitudinal	-0.07	0.12	0.27	1	0.60	0.00	(0.00, 0.32)
Mean age			0.60	3	0.62	0.00	(0.00, 0.69)
SD of age			0.59	3	0.62	0.00	(0.00, 0.49)
Proportion of male			0.12	3	0.95	0.00	(0.00, 0.39)

*S.E.: robust (sandwich) standard error; F: F statistic; d.f.: the first degree(s) of freedom for the F statistics; RESI: robust effect size index; Meta-RESI: the RESI used to quantify the effects from the meta-analysis model; CI: confidence interval; SD: standard deviation.

Table S9: The meta-analysis results for the RESI for sex on total white matter volume (WMV). The residual degrees of freedom are 52. The regression model is weighted by the inverse standard errors of the RESIs.

	Estimate	S.E.	F	d.f.	p-value	Meta-RESI	95% CI
Design: longitudinal	-0.10	0.12	0.70	1	0.41	0.00	(0.00, 0.42)
Mean age			0.10	3	0.96	0.00	(0.00, 0.48)
SD of age			0.13	3	0.94	0.00	(0.00, 0.43)
Proportion of male			0.28	3	0.84	0.00	(0.00, 0.48)

*S.E.: robust (sandwich) standard error; F: F statistic; d.f.: the first degree(s) of freedom for the F statistics; RESI: robust effect size index; Meta-RESI: the RESI used to quantify the effects from the meta-analysis model; CI: confidence interval; SD: standard deviation.

Table S10: The meta-analysis results for the RESI for sex on mean cortical thickness (CT). The residual degrees of freedom are 32. The regression model is weighted by the inverse standard errors of the RESIs.

	Estimate	S.E.	F	d.f.	p-value	Meta-RESI	95% CI
Design: longitudinal	-0.01	0.03	0.05	1	0.82	0.00	(0.00, 0.30)
Mean age			0.27	3	0.85	0.00	(0.00, 0.77)
SD of age			0.48	3	0.70	0.00	(0.00, 0.63)
Proportion of male			0.77	3	0.52	0.00	(0.00, 0.86)

*S.E.: robust (sandwich) standard error; F: F statistic; d.f.: the first degree(s) of freedom for the F statistics; RESI: robust effect size index; Meta-RESI: the RESI used to quantify the effects from the meta-analysis model; CI: confidence interval; SD: standard deviation.

Table S11: The descriptive summary of the non-brain measures in ABCD study used for the structural brain outcome analyses. The records with missing global and regional brain measures and the participants with missing baseline records are removed.

	**Baseline (N=11725)**	**2-year follow-up (N=8023)**	**4-year follow-up (N=3000)**	**Overall (N=22748)**
**Picture Vocabulary Test**				
Mean (SD)	52.3 (11.0)	49.7 (10.2)	50.1 (10.3)	51.1 (10.7)
Median [Min, Max]	51.0 [-12.0, 131]	49.0 [19.0, 88.0]	49.0 [19.0, 81.0]	50.0 [-12.0, 131]
Missing	748 (6.4%)	463 (5.8%)	135 (4.5%)	1346 (5.9%)
**Flanker Inhibitory Control and Attention Test**				
Mean (SD)	46.1 (9.13)	46.9 (9.64)	48.2 (10.6)	46.6 (9.54)
Median [Min, Max]	46.0 [10.0, 113]	46.0 [19.0, 81.0]	47.0 [19.0, 81.0]	46.0 [10.0, 113]
Missing	754 (6.4%)	989 (12.3%)	224 (7.5%)	1967 (8.6%)
**List Sorting Working Memory Test**				
Mean (SD)	49.5 (9.90)	48.2 (10.5)	51.1 (10.1)	49.8 (9.97)
Median [Min, Max]	48.0 [9.00, 108]	46.0 [19.0, 68.0]	51.0 [19.0, 81.0]	49.0 [9.00, 108]
Missing	790 (6.7%)	7966 (99.3%)	146 (4.9%)	8902 (39.1%)
**Dimensional Change Card Sort Test**				
Mean (SD)	47.5 (9.65)	47.6 (10.6)	47.5 (11.6)	47.5 (9.67)
Median [Min, Max]	46.0 [24.0, 132]	45.0 [26.0, 76.0]	45.0 [26.0, 81.0]	46.0 [24.0, 132]
Missing	751 (6.4%)	7974 (99.4%)	2923 (97.4%)	11648 (51.2%)
**Pattern Comparison Processing Speed Test**				
Mean (SD)	45.3 (14.4)	54.4 (13.7)	60.4 (13.8)	50.4 (15.2)
Median [Min, Max]	45.0 [-5.00, 100]	54.0 [19.0, 84.0]	61.0 [19.0, 81.0]	50.0 [-5.00, 100]
Missing	769 (6.6%)	1021 (12.7%)	229 (7.6%)	2019 (8.9%)
**Picture Sequence Memory Test**				
Mean (SD)	49.5 (11.0)	53.1 (11.4)	54.9 (13.6)	51.5 (11.7)
Median [Min, Max]	49.0 [10.0, 96.0]	52.0 [19.0, 81.0]	54.0 [19.0, 81.0]	50.0 [10.0, 96.0]
Missing	757 (6.5%)	396 (4.9%)	152 (5.1%)	1305 (5.7%)
**Oral Reading Recognition Test**				
Mean (SD)	49.4 (11.6)	48.7 (10.6)	51.4 (10.8)	49.4 (11.2)
Median [Min, Max]	47.0 [26.0, 142]	48.0 [24.0, 97.0]	51.0 [23.0, 81.0]	48.0 [23.0, 142]
Missing	759 (6.5%)	487 (6.1%)	153 (5.1%)	1399 (6.2%)
**Cognition Fluid Composite**				
Mean (SD)	45.8 (11.1)	44.9 (12.1)	47.4 (12.0)	45.8 (11.2)
Median [Min, Max]	45.0 [10.0, 128]	41.5 [24.0, 79.0]	47.5 [23.0, 73.0]	45.0 [10.0, 128]
Missing	961 (8.2%)	7971 (99.4%)	2950 (98.3%)	11882 (52.2%)
**Crystallized Composite**				
Mean (SD)	51.0 (11.2)	49.2 (10.7)	50.0 (11.3)	50.3 (11.1)
Median [Min, Max]	49.0 [8.00, 147]	48.0 [21.0, 100]	49.0 [22.0, 81.0]	49.0 [8.00, 147]
Missing	910 (7.8%)	1652 (20.6%)	2493 (83.1%)	5055 (22.2%)
**Cognition Total Composite Score**				
Mean (SD)	47.8 (11.2)	46.2 (10.4)	48.8 (12.3)	47.8 (11.2)
Median [Min, Max]	47.0 [5.00, 155]	45.5 [29.0, 71.0]	48.5 [24.0, 78.0]	47.0 [5.00, 155]
Missing	965 (8.2%)	7971 (99.4%)	2950 (98.3%)	11886 (52.3%)
**Total Problem CBCL Syndrome Scale**				
Mean (SD)	45.8 (11.4)	44.9 (11.3)	44.4 (11.5)	45.3 (11.4)
Median [Min, Max]	45.0 [24.0, 83.0]	45.0 [24.0, 88.0]	45.0 [24.0, 84.0]	45.0 [24.0, 88.0]
Missing	7 (0.1%)	1345 (16.8%)	23 (0.8%)	1375 (6.0%)
**Birth Weight (in oz)**				
Mean (SD)	112 (23.5)	113 (23.6)	112 (24.0)	112 (23.6)
Median [Min, Max]	115 [34.0, 236]	115 [34.0, 236]	115 [41.0, 200]	115 [34.0, 236]
Missing	1245 (10.6%)	762 (9.5%)	238 (7.9%)	2245 (9.9%)
**BMI**				
Mean (SD)	18.9 (3.96)	20.6 (4.53)	22.4 (4.81)	19.9 (4.46)
Median [Min, Max]	17.7 [13.5, 36.8]	19.4 [13.5, 36.9]	21.3 [13.5, 36.8]	18.8 [13.5, 36.9]
Missing	248 (2.1%)	140 (1.7%)	107 (3.6%)	495 (2.2%)
**Handedness**				
Right-handed	9312 (79.4%)	6368 (79.4%)	2448 (81.6%)	18128 (79.7%)
Not right-handed	2413 (20.6%)	1655 (20.6%)	552 (18.4%)	4620 (20.3%)

Table S12: The descriptive summary of the non-brain measures in the subset of ABCD study used for the functional connectivity (FC) outcome analyses. Only the participants with both baseline and 2-year follow-up are included.

	**Baseline** (***N*=1192**)	**2-year follow-up** (***N*=1192**)	**Overall** (***N*=2384**)
**Picture Vocabulary Test**			
Mean (SD)	52.8 (11.2)	50.2 (10.4)	51.5 (10.9)
Median [Min, Max]	52.0 [14.0, 112]	49.0 [19.0, 88.0]	50.0 [14.0, 112]
Missing	62 (5.2%)	75 (6.3%)	137 (5.7%)
**Flanker Inhibitory Control and Attention Test**			
Mean (SD)	46.0 (9.17)	46.9 (9.40)	46.5 (9.29)
Median [Min, Max]	45.5 [10.0, 113]	46.0 [19.0, 81.0]	46.0 [10.0, 113]
Missing	62 (5.2%)	130 (10.9%)	192 (8.1%)
**List Sorting Working Memory Test**			
Mean (SD)	50.1 (9.55)	52.5 (12.0)	50.1 (9.58)
Median [Min, Max]	49.0 [26.0, 91.0]	48.0 [34.0, 68.0]	49.0 [26.0, 91.0]
Missing	67 (5.6%)	1181 (99.1%)	1248 (52.3%)
**Dimensional Change Card Sort Test**			
Mean (SD)	47.9 (9.78)	47.1 (14.4)	47.9 (9.81)
Median [Min, Max]	46.0 [26.0, 98.0]	41.0 [33.0, 71.0]	46.0 [26.0, 98.0]
Missing	63 (5.3%)	1184 (99.3%)	1247 (52.3%)
**Pattern Comparison Processing Speed Test**			
Mean (SD)	45.9 (13.9)	54.4 (13.3)	50.0 (14.3)
Median [Min, Max]	46.0 [-2.00, 88.0]	54.0 [19.0, 81.0]	50.0 [-2.00, 88.0]
Missing	64 (5.4%)	137 (11.5%)	201 (8.4%)
**Picture Sequence Memory Test**			
Mean (SD)	49.8 (11.2)	53.6 (11.4)	51.7 (11.4)
Median [Min, Max]	49.0 [15.0, 96.0]	52.0 [19.0, 81.0]	51.0 [15.0, 96.0]
Missing	63 (5.3%)	66 (5.5%)	129 (5.4%)
**Oral Reading Recognition Test**			
Mean (SD)	49.6 (11.3)	49.4 (10.9)	49.5 (11.1)
Median [Min, Max]	48.0 [28.0, 118]	48.0 [24.0, 97.0]	48.0 [24.0, 118]
Missing	64 (5.4%)	78 (6.5%)	142 (6.0%)
**Cognition Fluid Composite**			
Mean (SD)	46.4 (11.2)	49.8 (17.9)	46.5 (11.3)
Median [Min, Max]	46.0 [20.0, 113]	47.0 [29.0, 79.0]	46.0 [20.0, 113]
Missing	81 (6.8%)	1182 (99.2%)	1263 (53.0%)
**Crystallized Composite**			
Mean (SD)	51.3 (11.2)	49.9 (10.9)	50.7 (11.1)
Median [Min, Max]	50.0 [22.0, 110]	49.0 [23.0, 100]	49.0 [22.0, 110]
Missing	80 (6.7%)	201 (16.9%)	281 (11.8%)
**Cognition Total Composite Score**			
Mean (SD)	48.4 (11.2)	50.7 (12.8)	48.4 (11.2)
Median [Min, Max]	48.0 [18.0, 127]	53.5 [29.0, 70.0]	48.0 [18.0, 127]
Missing	84 (7.0%)	1182 (99.2%)	1266 (53.1%)
**Total Problem CBCL Syndrome Scale**			
Mean (SD)	45.4 (11.1)	44.3 (11.0)	44.9 (11.1)
Median [Min, Max]	45.0 [24.0, 81.0]	44.5 [24.0, 78.0]	45.0 [24.0, 81.0]
Missing	0 (0%)	160 (13.4%)	160 (6.7%)
**Birth Weight**			
Mean (SD)	112 (23.8)	112 (23.8)	112 (23.8)
Median [Min, Max]	114 [35.0, 208]	114 [35.0, 208]	114 [35.0, 208]
Missing	121 (10.2%)	116 (9.7%)	237 (9.9%)
**BMI**			
Mean (SD)	18.0 (3.27)	19.5 (3.67)	18.7 (3.56)
Median [Min, Max]	17.1 [13.2, 30.7]	18.7 [13.2, 32.2]	17.9 [13.2, 32.2]
Missing	26 (2.2%)	21 (1.8%)	47 (2.0%)
**Handedness**			
Right-handed	968 (81.2%)	968 (81.2%)	1936 (81.2%)
Not right-handed	224 (18.8%)	224 (18.8%)	448 (18.8%)

Table S13: The estimated between- and within-subject parameters of the non-brain covariates on total gray matter volume (GMV), respectively, in the ABCD study. See Methods for more details about the estimation of the between- and within-subject effect parameters. The parameter estimates that are significantly different from zero are in bold. The intra-subject correlation of the outcome total GMV is estimated from the GEEs (generalized estimating equations) with an exchangeable correlation structure used for the modeling.

**Outcome**	**Non-brain covariate**	**Between-subject effect parameter (95% CI)**	**Within-subject effect parameter (95% CI)**	**Intra-subject correlation**
Total GMV	Crystallized Composite	**0.09** (0.08, 0.10)	-0.01 (-0.01, 0.00)	0.90
	Flanker Inhibitory Control and Attention	0.01 (-0.01, 0.02)	0.00 (0.00, 0.01)	0.91
	List Sorting Working Memory	0.06 (0.05, 0.07)	0.00 (-0.02, 0.01)	0.80
	Pattern Comparison Processing Speed	**0.01** (0.00, 0.01)	0.00 (-0.01, 0.00)	0.91
	Picture Sequence Memory	**0.01** (0.00, 0.02)	0.00 (0.00, 0.01)	0.92
	Picture Vocabulary	**0.07** (0.06, 0.08)	0.00 (0.00, 0.01)	0.91
	Oral Reading Recognition	**0.07** (0.06, 0.08)	**-0.01** (-0.01, 0.00)	0.91
	CBCL	**-0.04** (-0.05, -0.03)	-0.01 (-0.01, 0.00)	0.92
	BMI	**-0.13** (-0.16, -0.11)	-0.01 (-0.03, 0.01)	0.92

*CI: confidence interval; GMV: gray matter volume

3 Robust Effect Size Index (RESI) in Longitudinal Analysis

Standardized effect size (ES) indices are measures quantifying the strength of association between a variable and an outcome of interest. They are unaffected by the sample size and play an important role in power analyses, sample size planning, meta-analyses, and replicability[1–3]. Reporting ES estimates with confidence intervals (CIs) can simultaneously communicate the strength of evidence (sample size independent) as well as the precision in the observed evidence (sample size dependent).

We recently proposed a robust ES index (RESI) in the setting of cross-sectional data analysis [4, 5], which is a standardized parameter describing the deviation of the true parameter value from a reference value. It has several advantages over previously proposed indices [1, 6–9] because: it is widely applicable to many types of data since it is constructed from M-estimators, which are generally defined; it is robust to possible model misspecification as it can use a heteroskedasticity consistent covariance estimator [10]; it can accommodate the existence of nuisance covariates/parameters. We also provided a consistent estimator and a valid CI estimation method for the RESI [4, 5].

The correlation between observations makes the analysis of longitudinal data complicated. Generalized estimating equations (GEEs) [11], which estimate marginal associations, are commonly used in the analysis of longitudinal data. There are at least two advantages of GEEs: robustness to misspecification of the correlation structure of the repeatedly measured data; and computational simplicity. Technically, standardized ES indices designed for cross-sectional analysis can still be applied in longitudinal analysis, but the resulting standardized ES will incorporate any benefits of the longitudinal designs (i.e., the repeated measurements). The additional benefit of the longitudinal design introduces a systematic difference in the standardized ES estimates not only between longitudinal and cross-sectional data designs, but also between the longitudinal designs with different numbers of measurements per participant. Such a systematic difference compromises the comparability of the standardized ESs between studies with different designs. If we would like to compare the standardized ESs from studies studying similar scientific questions, but using different designs, it would be ideal to put them on the same "scale". To our knowledge, there is no ES index defined for longitudinal analysis models with this purpose.

In this section, we modify the RESI for longitudinal data analysis (with the modification called the "cross-sectional" RESI or CS-RESI) based on GEEs to make it comparable between longitudinal and cross-sectional studies and propose an estimator and CI estimation method for it. We conduct simulation studies to evaluate the consistency of the estimator, the nominal coverage of the CI estimation method, and the comparability between the CS-RESI in longitudinal analysis and the previously proposed RESI in an equivalent cross-sectional analysis.

3.1 RESI for generalized estimating equations

Let Y_{ij} denote the outcome variable and X_{ij} be a vector of length p explanatory variables (i.e., covariates) observed at time t_{ij} for observation $j = 1, \dots, n_i$ from $i = 1, \dots, N$ independent participants. Let $n = \sum_{i=1}^{N} n_i$ be the total number of measurements in the sample. The set of repeated measurements from participant i is a vector of length n_i, $Y_i = (Y_{i1}, Y_{i2}, \dots, Y_{in_i})$. We can assume the marginal mean model to be

$$\mathbb{E}(Y_{ij}|X_{ij}) = \mu_{ij}(\theta) = g^{-1}\left(X_{ij}^T\theta\right),$$

where $\mathbb{E}(Y_{ij}|X_{ij})$ is the expectation of Y_{ij} conditional on X_{ij} and g is a known link function [11]. $\theta = (\alpha, \beta) \in \Theta \subset \mathbb{R}^p$ are the model parameters, where $\alpha \in \mathbb{R}^{p_0}$ is a vector of nuisance parameters, $\beta \in \mathbb{R}^{p_1}$ is a vector of target parameter, and $p = p_0 + p_1$ which is the total number of parameters.

Let $V_i(\rho) = \mathrm{Var}(Y_i)$ denote the working covariance structure of Y_i, which depends on the marginal means and additional correlation parameter ρ. The estimating function used by GEEs for θ is given by

$$U_\theta(\theta, \rho) = \sum_{i=1}^{N} D_i^T V_i^{-1}(\rho)(Y_i - \mu_i), \tag{1}$$

where $D_i = (\partial\mu_i/\partial\theta)^T$ and $\mu_i = (\mu_{i1}, \mu_{i2}, \dots, \mu_{in_i})$, is the marginal mean for participant i for the n_i observations [11].

For a given value of ρ, the solution to $U_\theta(\hat{\theta}, \rho) = 0$ defines an estimator $\hat{\theta}$. Under mild regularity conditions [11], the estimator for θ obtained from (1) is consistent and

$$\sqrt{N}(\hat{\theta} - \theta) \xrightarrow{d} \text{MVN}_p\left(0, \Sigma_\theta\right).$$

where

$$\begin{aligned} \Sigma_\theta &= A^{-1}BA^{-1} \\ A &= \lim_{N\to\infty} N^{-1}\sum_{i=1}^{N} D_i^T V_i^{-1} D_i \\ B &= \lim_{N\to\infty} N^{-1}\sum_{i=1}^{N} D_i^T V_i^{-1} \text{Cov}(Y_i) V_i^{-1} D_i \end{aligned} \tag{2}$$

If the correlation model is correctly specified, $\text{Cov}(Y_i) = V_i$, then $\Sigma_\theta = A^{-1}$.

When Σ_θ is known, the Wald-type statistic testing the null hypothesis $H_0 : \beta = \beta_0$, where β_0 is the reference values, follows a chi-squared distribution with p_1 degrees of freedom and non-centrality parameter (NCP) $N(\beta - \beta_0)^T \Sigma_\beta^{-1}(\theta)(\beta - \beta_0)$,

$$T^2 = N(\hat{\beta} - \beta_0)^T \Sigma_\beta^{-1}(\theta)(\hat{\beta} - \beta_0) \sim \chi^2_{p_1}\left(N(\beta - \beta_0)^T \Sigma_\beta^{-1}(\theta)(\beta - \beta_0)\right),$$

where $\hat{\beta}$ is the estimator for β and $\Sigma_\beta(\theta)$ is the asymptotic covariance matrix of $\sqrt{N}(\hat{\beta} - \beta)$.

3.1.1 Longitudinal RESI (L-RESI)

The original definition of the RESI for cross-sectional analysis [4] is still applicable here, and is defined as the square root of the component of the NCP that is due to the deviation of β from the reference values,

$$S = \sqrt{(\beta - \beta_0)^T \Sigma_\beta^{-1}(\beta - \beta_0)}. \tag{3}$$

We also defined a signed version of the RESI for univariate parameters β [12]

$$S_{\text{sgn}} = \beta / \Sigma_\beta^{1/2}, \tag{4}$$

where $\Sigma_\beta \in \mathbb{R}$. The absolute value of S_{sgn} is equal to S.

The previously defined estimators for (3) and (4) are consistent in this context by the theory described in previous work [4, 5]:

$$\begin{aligned} \hat{S} &= \left(\max\left\{0, \frac{\hat{T}^2 - p_1}{N}\right\}\right)^{\frac{1}{2}} \text{ and} \\ \hat{S}_{\text{sgn}} &= \frac{\hat{T}}{\sqrt{N}}. \end{aligned}$$

The RESI is independent of the sample size N but dependent on the within-subject correlation. This can be seen through the dependence of (3) and (4) on $\text{Cov}(Y_i)$ through (2). The RESI in equation (3) quantifies the standardized ES over the entire follow-up, therefore is influenced by the study design such as the number of measurements, length of follow-up time, and correlation structure. Throughout this document, we refer to the RESI defined in the setting of cross-sectional analysis but applied in longitudinal analysis as longitudinal RESI or L-RESI to indicate that it implicitly incorporates the information of the longitudinal design. This is different from the main paper, where we use the term "RESI" for this value regardless of cross-sectional or longitudinal analysis.

3.1.2 "Cross-sectional" RESI (CS-RESI) in longitudinal analysis

The systematic difference in the standardized ESs between cross-sectional and longitudinal studies makes comparing and aggregating cross-sectional and longitudinal studies more challenging in meta-analysis. In order to allow more consistent comparisons of standardized ES estimates across cross-sectional and longitudinal studies, we modify the definition of the RESI to let it represent the standardized ES as if the study was conducted cross-sectionally, but using the longitudinal data. As β quantifies the marginal effect, it is equivalent in cross-sectional and longitudinal studies. Thus, only the variance in (3) is different between cross-sectional and longitudinal studies, so the target parameter is

$$S_{cs} = \sqrt{(\beta - \beta_0)^T \Sigma_{\beta_{cs}}^{-1} (\beta - \beta_0)}, \tag{5}$$

where $\Sigma_{\beta_{cs}}$ is the asymptotic covariance matrix of $\sqrt{N}(\hat{\beta} - \beta)$ from an equivalent cross-sectional study. We refer to this as "CS-RESI" in a longitudinal study.

To relate the equivalent cross-sectional study to the longitudinal study, we assume

$$L_{cs} = RL, \tag{6}$$

where $L = [Y,\ X]$ is an $n \times (p+1)$ matrix of the full longitudinal data including n_i measurements for each participant i, L_{cs} is an $N \times (p+1)$ matrix of covariates and outcome Y, and R is an $N \times n$ random block sampling matrix that samples one row for each participant in the longitudinal study with equal probability. We assume that R is independent of L, so that the data are missing completely at random.

If the mean model is correctly specified, estimation of the CS-RESI for GEEs boils down to obtaining a consistent estimator for $\Sigma_{\beta_{cs}}$ using the longitudinal data. For identity link regression models, we propose the use of the estimator

$$\hat{\Sigma}_{\beta_{cs}} = (N^{-1}X^TWX)^{-1}(N^{-1}X^TW\hat{\Delta}WX)(N^{-1}X^TWX)^{-1} := \hat{A}_{cs}^{-1}\hat{B}_{cs}\hat{A}_{cs}^{-1}, \tag{7}$$

where W is a diagonal matrix that weights each observation by the inverse of the number of measurements for each participant, $\hat{\Delta}$ is a diagonal matrix with elements $\hat{\Delta}_{ii} = (Y_i - X_i\hat{\beta})^2/(1 - H_{ii})^2$ and H_{ii} is the i-th diagonal element of the hat matrix [10].

Theorem 1. *Let all objects be as defined above and assume that for all $a, b = 1, \ldots, p_1$, $\mathrm{Cov}\left(\hat{\epsilon}_{ij}^2, \hat{\epsilon}_{ik}^2\right)(x_{ija}x_{ijb})(x_{ika}x_{ikb})$ and n_i are bounded for all i, $j, k = 1, \ldots, n_i$. Then the estimator*

$$\hat{S} = \left(\max\left\{0, \frac{T_{cs}^2 - p_1}{N}\right\}\right)^{\frac{1}{2}}, \tag{8}$$

is consistent for (5), where $T_{cs}^2 = N(\hat{\beta} - \beta_0)^T \hat{\Sigma}_{\beta_{cs}}^{-1}(\hat{\beta} - \beta_0)$, and $\hat{\beta}$ is the estimator for β from a GEE.

Proof. Since the marginal parameters are estimated in GEEs using identify link function, the parameter estimates are consistent for the same parameter in the cross-sectional and longitudinal models. Therefore, the estimator (8) is a consistent estimator for the CS-RESI (5) as long as

$$\hat{\Sigma}_{\beta_{cs}} \xrightarrow{p} \Sigma_{\beta_{cs}},$$

where

$$\Sigma_{\beta_{cs}} = \lim_{N \to \infty} (N^{-1}X^TWX)^{-1}(N^{-1}X^TW\Delta WX)(N^{-1}X^TWX)^{-1} := A_{cs}^{-1}B_{cs}A_{cs}^{-1}.$$

It is sufficient to show that $\hat{A}_{cs} \xrightarrow{p} A_{cs}$ and $\hat{B}_{cs} \xrightarrow{p} B_{cs}$ then $\hat{\Sigma}_{\beta_{cs}} \xrightarrow{p} \Sigma_{\beta_{cs}}$ will follow by Slutsky's theorem.

The element on the a-th row and b-th column of B_{cs} is

$$\begin{aligned} B_{cs,(a,b)} &= \lim_{N \to \infty} \frac{1}{N} \mathbb{E}_R \left(\sum_{i=1}^{N} \sum_{j=1}^{n_i} R_{ij} x_{ija} x_{ijb} \sigma_{ij}^2 \right) \\ &= \lim_{N \to \infty} \frac{1}{N} \sum_{i=1}^{N} \frac{1}{n_i} \sum_{j=1}^{n_i} x_{ija} x_{ijb} \sigma_{ij}^2. \end{aligned}$$

$\hat{B}_{cs,(a,b)}$ converges in probability if it is asymptotically unbiased and $\text{Var}(\hat{B}_{a,b}) \to 0$ as $N \to \infty$ for a, $b = 1, \ldots, p_1$. The corresponding element of $\hat{B}_{cs}$ is $\frac{1}{N}\sum_{i=1}^{N}\frac{1}{n_i}\sum_{j=1}^{n_i} x_{ija}x_{ijb}\hat{\epsilon}_{ij}^2$, where x_{ija} is the value of the a-th covariates (including the intercept) of the j-th observation from the i-th participant. The asymptotic unbiasedness of $\hat{B}_{cs,(a,b)}$ follows because $\hat{\epsilon}_{ij}^2 = \frac{(Y_{ij}-X_{ij}\hat{\beta})^2}{1-h_{ij,ij}}$ is unbiased estimator for σ_{ij}^2. The variance of $\hat{B}_{cs,(a,b)}$ is

$$\begin{aligned}
\text{Var}(\hat{B}_{cs,(a,b)}) &= \text{Var}\left(\frac{1}{N}\sum_{i=1}^{N}\sum_{j=1}^{n_i}\frac{1}{n_i}x_{ija}x_{ijb}\hat{\epsilon}_{ij}^2\right) \\
&= \frac{1}{N^2}\sum_{i=1}^{N}\frac{1}{n_i^2}\sum_{j=1}^{n_i}\sum_{k=1}^{n_i}(x_{ija}x_{ijb})(x_{ika}x_{ikb})\text{Cov}\left(\hat{\epsilon}_{ij}^2, \hat{\epsilon}_{ik}^2\right) \\
&= \frac{1}{N^2}\times\sum_{i=1}^{N}\frac{1}{n_i^2}\left[\sum_{j=1}^{n_i}(x_{ija}x_{ijb})^2\text{Var}(\hat{\epsilon}_{ij}^2) + 2\sum_{j\leq k}\text{Cov}\left(\hat{\epsilon}_{ij}^2, \hat{\epsilon}_{ik}^2\right)(x_{ija}x_{ijb})(x_{ika}x_{ikb})\right] \\
&= \frac{1}{N^2}\mathcal{O}\left(\sum_{i=1}^{N}\frac{1}{n_i}\right) + \frac{2}{N^2}\mathcal{O}\left(\sum_{i=1}^{N}\frac{1}{n_i}\right) \\
&= \mathcal{O}\left(\frac{1}{N^2}\sum_{i=1}^{N}\frac{1}{n_i}\right)
\end{aligned}$$

thus, $\text{Var}(\hat{B}_{cs,(a,b)}) \to 0$ as $N \to \infty$.

Similarly, the element on the a-th row and b-th column of $\hat{A}_{cs}$ and A_{cs} are

$$\hat{A}_{cs,(a,b)} = \frac{1}{N}\sum_{i=1}^{N}\frac{1}{n_i}\sum_{j=1}^{n_i}x_{ija}x_{ijb}$$

and

$$\begin{aligned}
A_{cs,(a,b)} &= \frac{1}{N}\sum_{i=1}^{N}\sum_{j=1}^{n_i}\mathbb{E}_R(R_{ij})x_{ija}x_{ijb} \\
&= \frac{1}{N}\sum_{i=1}^{N}\frac{1}{n_i}\sum_{j=1}^{n_i}x_{ija}x_{ijb}
\end{aligned}$$

Thus, for given $\boldsymbol{X}$, $Var(\hat{A}_{cs,(a,b)}) = 0$ and $\mathbb{E}(\hat{A}_{cs,(a,b)}) - A_{cs,(a,b)} \to 0$, thus we have $\hat{A}_{cs} \xrightarrow{p} A_{cs}$. Then $\hat{\Sigma}_{\beta_{cs}} \xrightarrow{p} \Sigma_{cs}$ by Slutsky's theorem, which completes the proof.

□

The CS-RESI estimate assumes that the parameters estimated in the cross-sectional and longitudinal models are equivalent. This is violated when the actual between- and within-subject effects are not equal and the longitudinal analysis model (either linear mixed model (LMM) or GEE) assumes a non-independence working covariance structure (see Section 5.3).

3.1.3 Confidence interval construction for CS-RESI

As shown in our previous work, the distribution of the test statistic may deviate from the theoretical chi-square or F distributions when an estimator is used for the covariance and/or the design matrix is random [5]. In these situations, the confidence intervals (CIs) constructed based on the theoretical distributions will fail to provide nominal coverage. Bootstrapping can be used to approximate the sampling distribution of the RESI estimators. We consider the standard nonparametric bootstrap by sampling the participants with replacement r times and estimating the corresponding L-RESI and CS-RESI for each resampled data. The lower and upper $\alpha/2 \times 100$ percentiles of the r estimated RESIs are the bootstrapped lower and upper bounds of the $(1-\alpha)\times 100$ bootstrap CI for the estimated L-RESI or CS-RESI.

3.2 Simulation Studies

In the previous sections, we define the L-RESI and CS-RESI, propose corresponding estimators, and the non-parametric bootstrap for CI construction. Here, we use simulations to evaluate the influence of fixed/random covariates, and model misspecification on the performance of the estimators and CIs.

3.2.1 Simulation setup

We simulate longitudinal data for each participant using a linear model $Y_i = \beta_0 + \beta_g \times \text{group} + \beta_t \times \text{time} + \epsilon_i$, which contains a time-independent binary variable "group" and a time-dependent variable "time" across 5 visits, for 1,000 independent simulations. To simulate the within-subject correlation, we specify $\text{Cov}(\epsilon_i)$ to have either exchangeable or first-order autoregressive (AR-1) correlation structure. When using the exchangeable correlation structure,

$$\text{Cov}(\epsilon_i) = \begin{bmatrix} 1 & 0.5 & \cdots & 0.5 \\ 0.5 & 1 & \cdots & 0.5 \\ \vdots & & \ddots & \vdots \\ 0.5 & 0.5 & \dots & 1 \end{bmatrix},$$

and when using the AR-1 structure,

$$\text{Cov}(\epsilon_i) = \begin{bmatrix} 1 & 0.5 & 0.5^2 & 0.5^3 & 0.5^4 \\ 0.5 & 1 & & \cdots & 0.5^3 \\ \vdots & & & \ddots & \vdots \\ 0.5^4 & 0.5^3 & 0.5^2 & \dots & 1 \end{bmatrix}.$$

We vary the sample size $N \in \{50, 250, 500\}$ across three different true L-RESI values $S \in \{0, 0.5, 1\}$. The true L-RESIs of the group variable and the time variable are specified to be the same in each scenario. The true CS-RESI values are calculated accordingly. To show the influence of experimental and observational designs where the covariates are fixed or random, the values of the covariates are generated in two ways: (1) when being fixed, $\pi = 0.5$ and the random ceiling($N\pi$) of the N individual participants have their group variable with value 1 and the remaining have value 0, and everyone has 5 different observations at timepoints $= \{0, 1, \cdots, 4\}$; (2) when being random, group $\sim$ Bernoulli(π) is sampled from a Bernoulli distribution with parameter $\pi = 0.5$ for each participant, and everyone has 5 observations at 5 timepoints randomly chosen in the range of 0 to 4. The working correlation structure is specified to be "exchangeable" or "AR1", regardless of the true correlation structure to evaluate the influence of model misspecification on the performance of the estimators and CIs. For each bootstrap CI, 1,000 bootstraps are used.

To evaluate the consistency of the CS-RESI between the longitudinal analysis and its equivalent cross-sectional analysis, we use block sampling to create the equivalent artificial cross-sectional sample by randomly selecting one observation from each participant in each of the simulated longitudinal datasets. In each of the simulations, the CS-RESI is estimated using the GEE model in the longitudinal data and the regular RESI designed for cross-sectional studies is estimated using a linear model with the same model formula as the GEE in the equivalent cross-sectional data. The mean difference between the CS-RESI from the longitudinal data and the regular RESI from the cross-sectional data (defined as the CS-RESI in the longitudinal data minus the regular RESI in the cross-sectional data) is estimated across the simulations. This mean difference should approach zero at large sample sizes if CS-RESI has the desired comparability.

3.2.2 Simulation results

Simulations are performed to assess the bias of the estimators and the coverage of the CIs. We consider different cases where there are random/fixed covariates and/or misspecification of the model correlation structure.

In small samples ($N = 50$), the estimators for the CS-RESI and the longitudinal RESI are both slightly biased for both the group and time variables, but the bias goes to zero as sample size increases

(Figure S5 and S6). The estimators for the CS-RESI and L-RESI show consistency in all scenarios, including when the correlation structure is mistakenly specified (Figure S5B and C; Figure S6B and C). The variable randomness doesn't show an influence on the estimator biasedness.

The bootstrap CI provides nominal coverage for the CS-RESI and L-RESI under all scenarios when the models are correctly specified (Figure S7A and D; S8A and D). When the models are misspecified (Figure S7B and C; S8B and C), the CI sometimes provides coverage lower than the nominal level, but the coverages approach the nominal level as sample size increases. The variable randomness doesn't show an influence on the coverage of bootstrap CI.

As to the consistency/comparability of the CS-RESI in the longitudinal study to the regular RESI in the equivalent cross-sectional study, Figure S9 shows the mean difference between them across all simulations. The difference is defined as CS-RESI minus regular RESI. In small samples ($N = 50$), the CS-RESI in longitudinal data slightly overestimates the effect of the group variable and underestimates the effect of the time variable in cross-sectional studies when $S = 0$, but the mean difference approaches zero in large sample sizes. When $S \neq 0$ and $N = 50$, the CS-RESI tends to overestimate the effect of both the group and time variables, but the mean difference goes to zero in large sample sizes. The results indicate that the CS-RESI in longitudinal studies essentially estimates the same thing as if the studies are conducted cross-sectionally and the consistency is not affected by model misspecification and variable randomness.

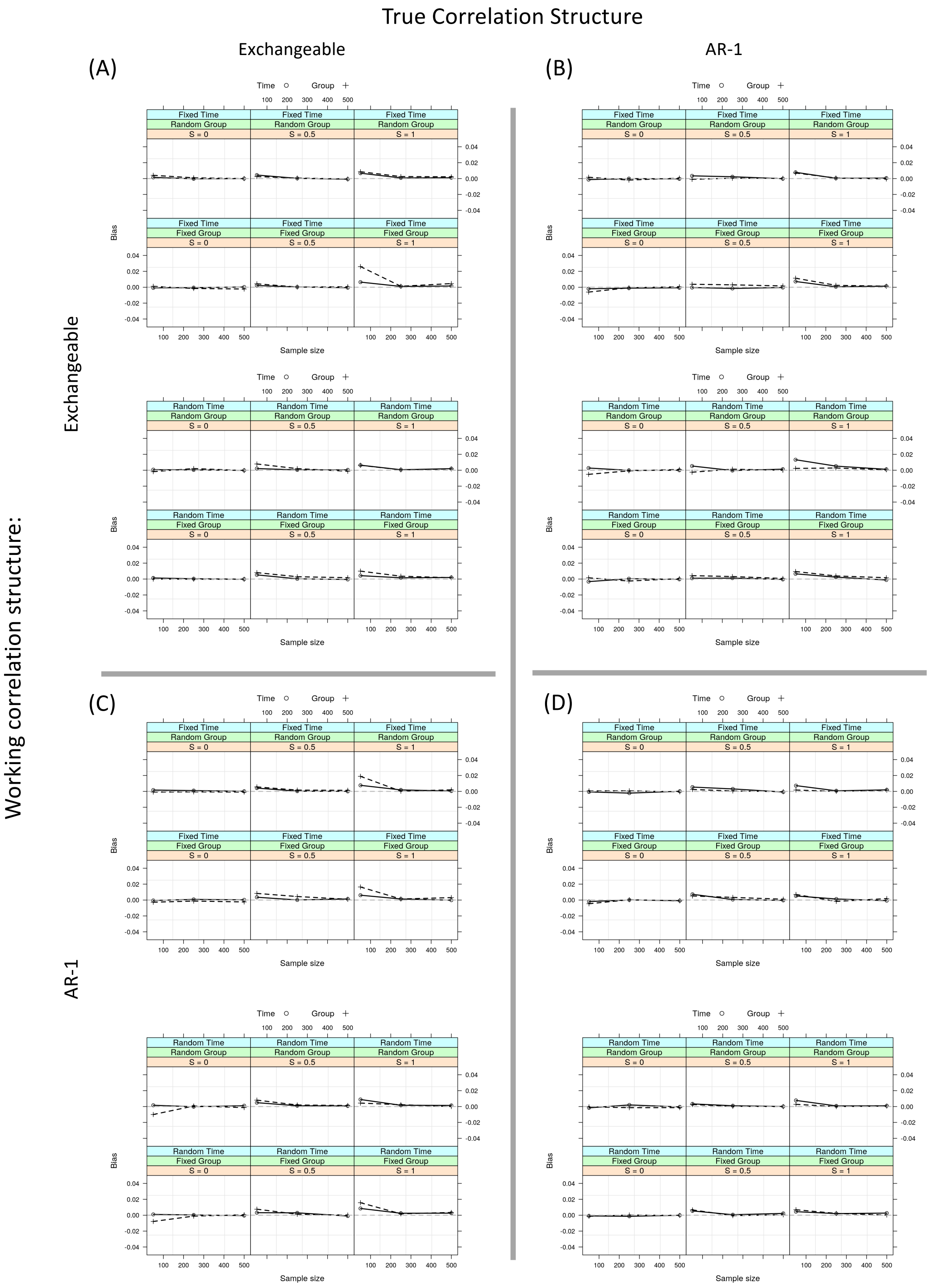

Figure S5: The bias of the CS-RESI estimator for both group and time variables under the simulation settings described above. The bias of the CS-RESI estimator goes to zero as sample size increases in all scenarios, indicating it is an asymptotic unbiased estimator. S denotes the L-RESI, which is specified the same for both the group and time variables; the true values of CS-RESI are calculated accordingly in each scenario.

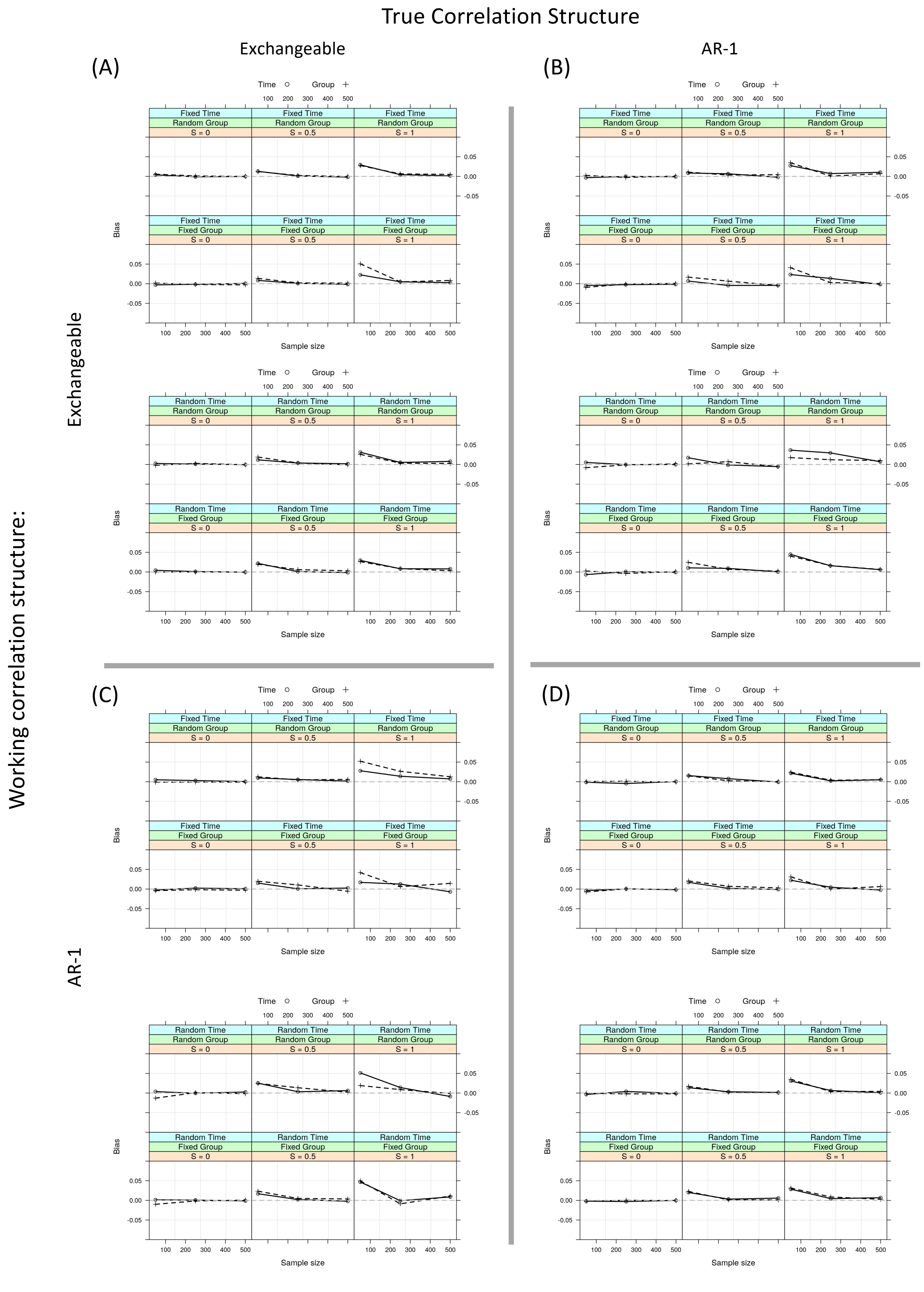

Figure S6: The bias of the L-RESI estimator for both group and time variables under the simulation settings described above. The bias of the L-RESI estimator goes to zero as sample size increases in all scenarios, indicating it's an asymptotic unbiased estimator. S denotes the longitudinal RESI, which is specified the same for both the group and time variables.

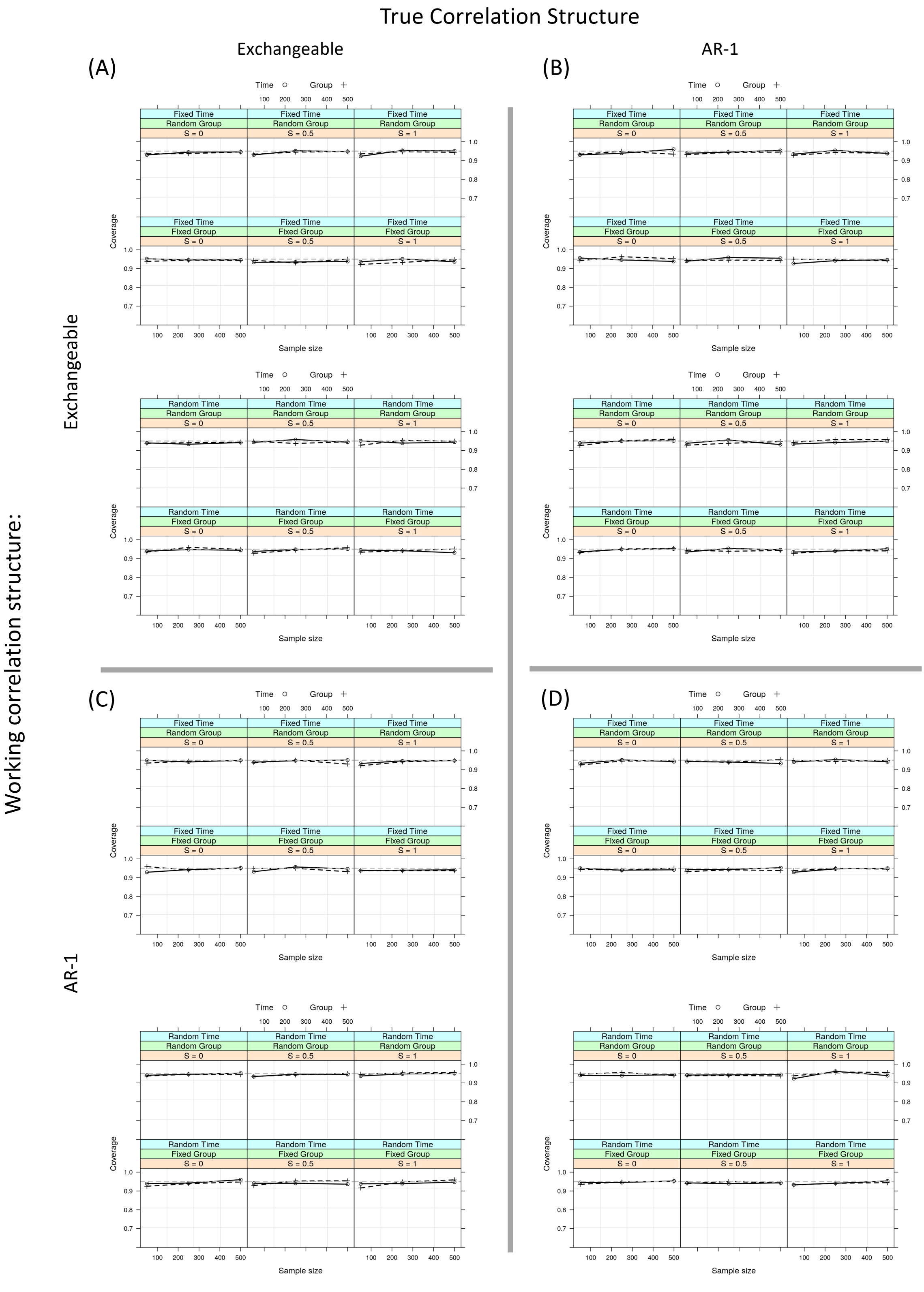

Figure S7: The coverage of bootstrap CI for the CS-RESI estimator under the simulation settings described above. The bootstrap CI asymptotically provides nominal coverage for the CS-RESI under all scenarios even when the models are misspecified. S denotes the L-RESI, which is specified the same for both the group and time variables; the true values of CS-RESI are calculated accordingly in each scenario.

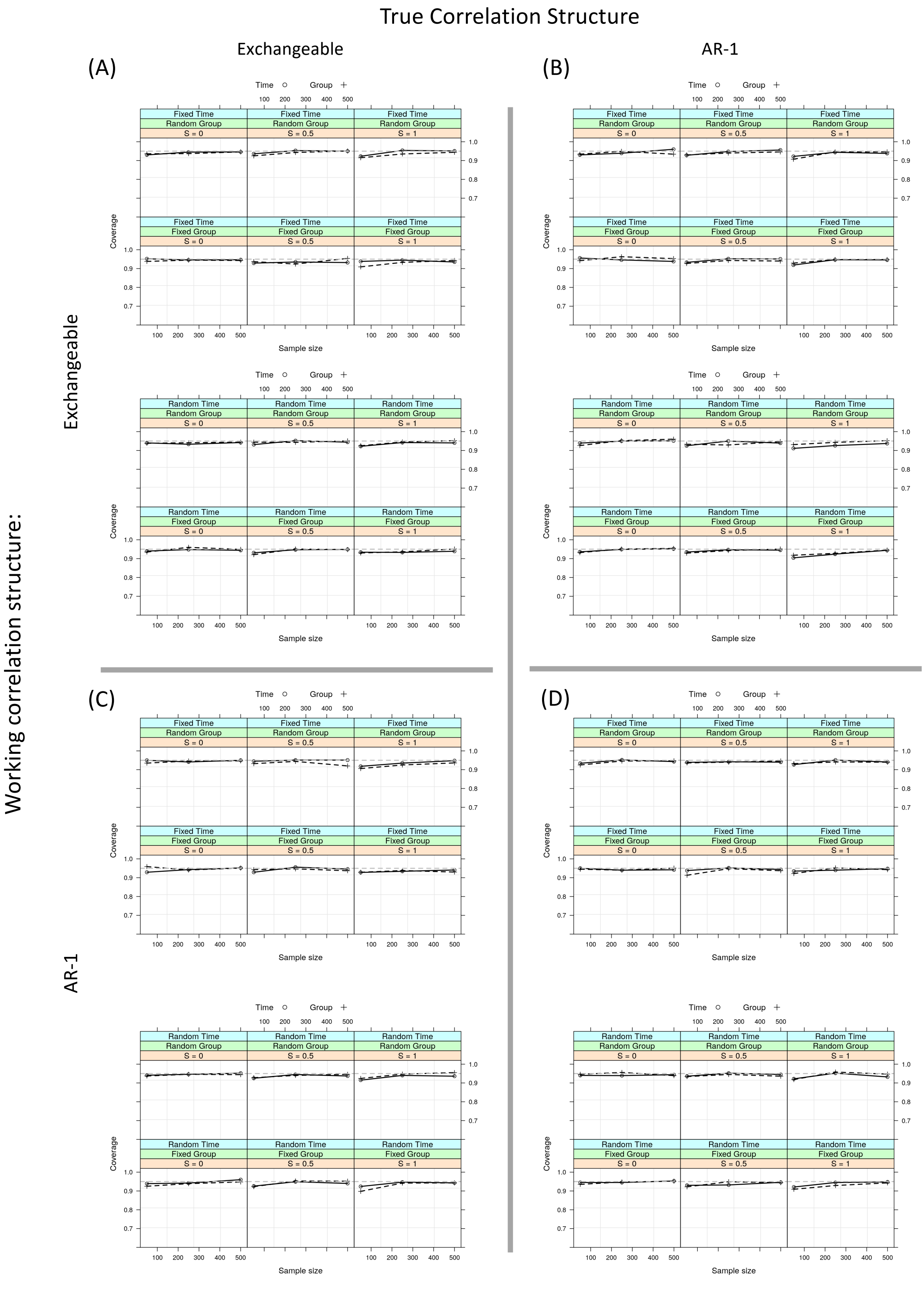

Figure S8: The coverage of bootstrap CI for the L-RESI estimator under the simulation settings described above. The bootstrap CI asymptotically provides nominal coverage for the L-RESI and under all scenarios even when the models are misspecified. S denotes the L-RESI, which is specified the same for both the group and time variables.

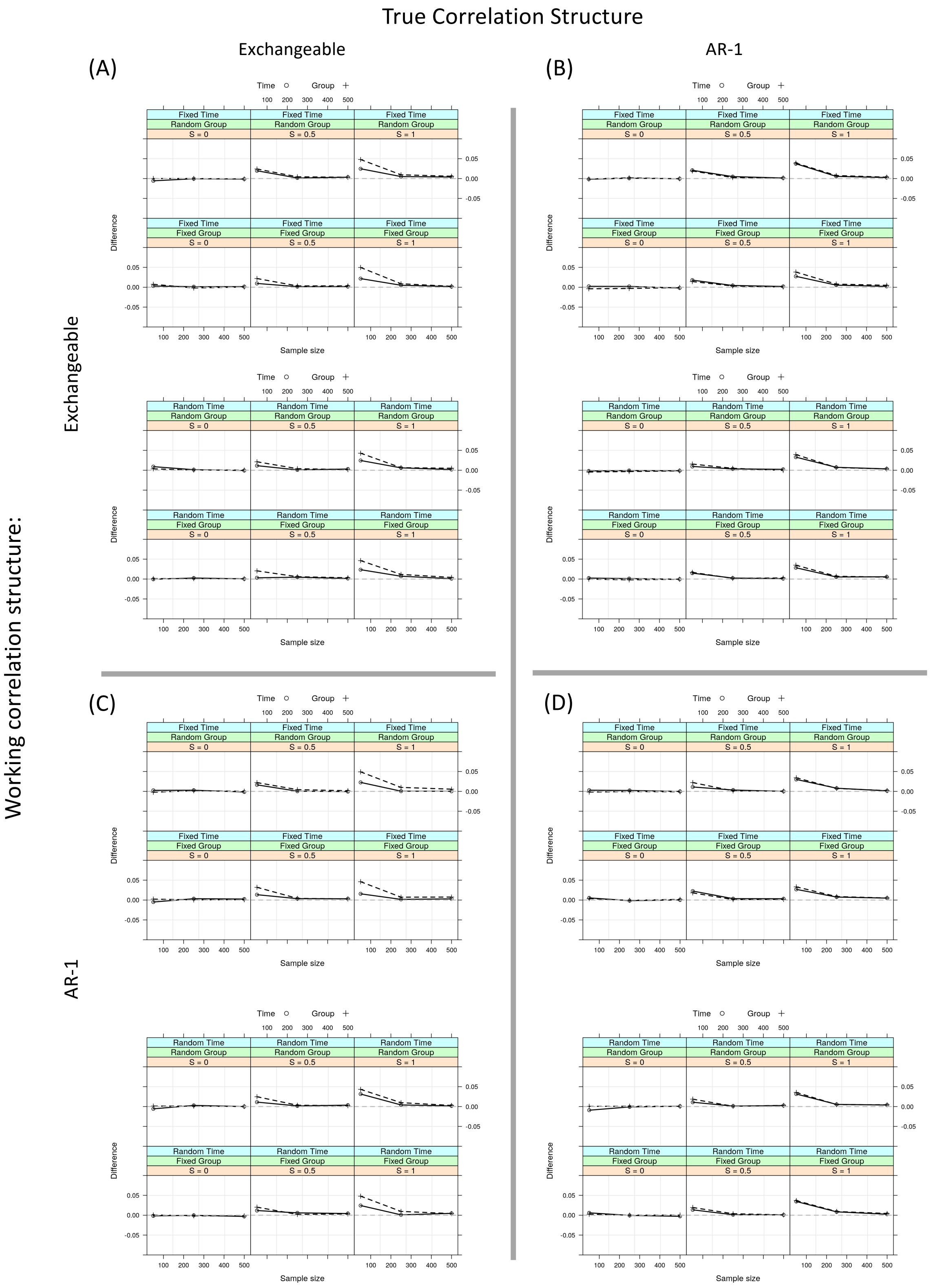

Figure S9: The mean difference between the CS-RESI in longitudinal studies and the regular RESI in the equivalent cross-sectional studies. The mean difference approaches zero in all scenarios including when the models are misspecified, indicating the consistency/comparability between the CS-RESI in longitudinal studies and the regular RESI in cross-sectional studies. S denotes the longitudinal RESI, which is specified the same for both the group and time variables.

3.3 Comparison of L-RESI and CS-RESI quantifies the benefit of longitudinal design

While the systematic difference in the standardized ES between cross-sectional and longitudinal study designs compromises the comparability of standardized ESs between studies, it is also a feature that we can leverage to quantify the benefit of using a longitudinal design in a single study. With this goal in mind, we compare the difference between the L-RESI and CS-RESI in each of the longitudinal studies from the LBCC analyzed in the main paper to quantify the benefit of using the longitudinal study design.

To demonstrate the difference in standardized ES by study design and validate the CS-RESI estimator, we use non-parametric bootstrapping to create longitudinal bootstrap samples by randomly sampling participants with replacement (1,000 bootstraps per sample size) in each of the 16 longitudinal neuroimaging datasets we use in the main paper. In this analysis and subsequent bootstrap analyses, the full dataset represents the unknown population, and bootstrap replicates represent random samples from the population. At a given sample size, after obtaining a longitudinal bootstrap sample (including all measurements from the selected participants), we create an equivalent cross-sectional version of the longitudinal bootstrap sample by randomly selecting one measurement from each selected participant. In each replicate, we fit the same GEEs and linear regression models of total GMV we use in the main paper to the longitudinal and cross-sectional bootstrap samples, and quantify the effects of age and sex on total GMV. L-RESI and CS-RESI are computed in the longitudinal bootstrap samples and compared to the RESI computed in the cross-sectional bootstrap samples. The differences of the RESI estimated in the cross-sectional sample to the CS-RESI and L-RESI estimated in the longitudinal sample are calculated in each replicate, and the mean differences are derived across the replicates. All site effects are removed using LongComBat before performing the bootstrap analysis.

When comparing the L-RESI estimated in the longitudinal samples to the RESI estimated in cross-sectional samples, the longitudinal standardized ESs for age and sex are typically larger (Figure S10a). The positive differences represent the amount of benefit of using longitudinal designs to quantify the age or sex effect for the two studies shown (ADNI and GUSTO). Age shows an increase of 0.16 in GUSTO and 0.25 in ADNI, while sex shows an expectedly smaller increase of 0.02 in GUSTO and ADNI (other studies shown in Figure S11). The difference we note in longitudinal standardized ESs is not restricted to the L-RESI, as Cohen's d for sex computed in a longitudinal study also shows the same slightly positive estimate (Figure S10a). In contrast, the mean difference between the CS-RESI computed in the longitudinal samples and the RESI computed in the cross-sectional samples approaches zero as the sample size increases (Figure S10b), indicating that the systematic difference in the standardized ESs between longitudinal and cross-sectional datasets is removed. As a further comparison, we repeat the meta-analyses in the main paper on the standardized ESs from all studies but now use the CS-RESIs to represent the standardized ESs in longitudinal studies, demonstrating a reduction in the design effect (Tab. S14, S15, S16, and S17).

Table S14: Meta-analysis results for the RESI for age on total gray matter volume (GMV). The residual degrees of freedom are 52. The regression model is weighted by the inverse standard errors of the L-RESIs. For cross-sectional studies, the outcome is RESI; for longitudinal studies, the outcome is CS-RESI.

	Estimate	S.E.	F	d.f.	p-value	Meta-RESI	95% CI
Design: Longitudinal	0.02	0.08	0.06	1	0.81	0.00	(0.00, 0.26)
Mean age			3.91	3	0.01	0.36	(0.18, 1.02)
SD of age			20.23	3	<0.01	0.94	(0.58, 1.72)
Skewness of age			0.77	3	0.52	0.00	(0.00, 0.61)

*S.E.: robust (sandwich) standard error; F: F statistic; d.f.: the first degree(s) of freedom for the F statistics; RESI: robust effect size index; L-RESI: longitudinal RESI; CS-RESI: cross-sectional RESI; Meta-RESI: the L-RESI used to quantify the effects from the meta-analysis model; CI: confidence interval; SD: standard deviation.

Table S15: Meta-analysis results for the RESI for age on total subcortical gray matter volume (sGMV). The residual degrees of freedom are 52. The regression model is weighted by the inverse standard errors of the RESIs. For cross-sectional studies, the outcome is RESI; for longitudinal studies, the outcome is CS-RESI.

	Estimate	S.E.	F	d.f.	p-value	Meta-RESI	95% CI
Design: Longitudinal	-0.03	0.07	0.20	1	0.65	0.00	(0.00, 0.3)
Mean age			1.10	3	0.36	0.05	(0.00, 0.77)
SD of age			7.09	3	<0.01	0.53	(0.06, 1.11)
Skewness of age			0.17	3	0.91	0.00	(0.00, 0.47)

*S.E.: robust (sandwich) standard error; F: F statistic; d.f.: the first degree(s) of freedom for the F statistics; RESI: robust effect size index; L-RESI: longitudinal RESI; CS-RESI: cross-sectional RESI; Meta-RESI: the L-RESI used to quantify the effects from the meta-analysis model; CI: confidence interval; SD: standard deviation.

Table S16: Meta-analysis results for the RESI for age on total white matter volume (WMV). The residual degrees of freedom are 52. The regression model is weighted by the inverse standard errors of the RESIs. For cross-sectional studies, the outcome is RESI; for longitudinal studies, the outcome is CS-RESI.

	Estimate	S.E.	F	d.f.	p-value	Meta-RESI	95% CI
Design: Longitudinal	0.00	0.06	0.00	1	0.95	0.00	(0.00, 0.28)
Mean age			4.88	3	<0.01	0.42	(0.21, 1.23)
SD of age			3.36	3	0.03	0.33	(0.00, 0.92)
Skewness of age			0.46	3	0.71	0.00	(0.00, 0.60)

*S.E.: robust (sandwich) standard error; F: F statistic; d.f.: the first degree(s) of freedom for the F statistics; RESI: robust effect size index; L-RESI: longitudinal RESI; CS-RESI: cross-sectional RESI; Meta-RESI: the L-RESI used to quantify the effects from the meta-analysis model; CI: confidence interval; SD: standard deviation.

Table S17: Meta-analysis results for the RESI for age on mean cortical thickness (CT). The residual degrees of freedom are 32. The regression model is weighted by the inverse standard errors of the RESIs. For cross-sectional studies, the outcome is RESI; for longitudinal studies, the outcome is CS-RESI.

	Estimate	S.E.	F	d.f.	p-value	Meta-RESI	95% CI
Design: Longitudinal	0.05	0.17	0.08	1	0.79	0.00	(0.00, 0.50)
Mean age			1.84	3	0.16	0.22	(0.00, 1.27)
SD of age			4.11	3	0.01	0.45	(0.00, 1.44)
Skewness of age			0.05	3	0.99	0.00	(0.00, 0.69)

*S.E.: robust (sandwich) standard error; F: F statistic; d.f.: the first degree(s) of freedom for the F statistics; RESI: robust effect size index; L-RESI: longitudinal RESI; CS-RESI: cross-sectional RESI; Meta-RESI: the L-RESI used to quantify the effects from the meta-analysis model; CI: confidence interval; SD: standard deviation.

The benefit of conducting the longitudinal design can be quantified in each study by comparing the CS-RESI estimates with the L-RESI estimates (Figure S10c; Figure S12 and Figure S13). Longitudinal designs result in a 0.31 average increase in the standardized ES for the total GMV-age association among the 16 longitudinal neuroimaging datasets (Table S3), corresponding to a moderate increase in standardized ES [4]. Notably, in some studies (e.g., POND and SCZIowa), the L-RESI is smaller than the CS-RESI, which implies that they lose efficiency by using longitudinal designs. We investigate this counterintuitive result further in our subsequent analyses of cognitive variables in the ABCD study in the main paper.

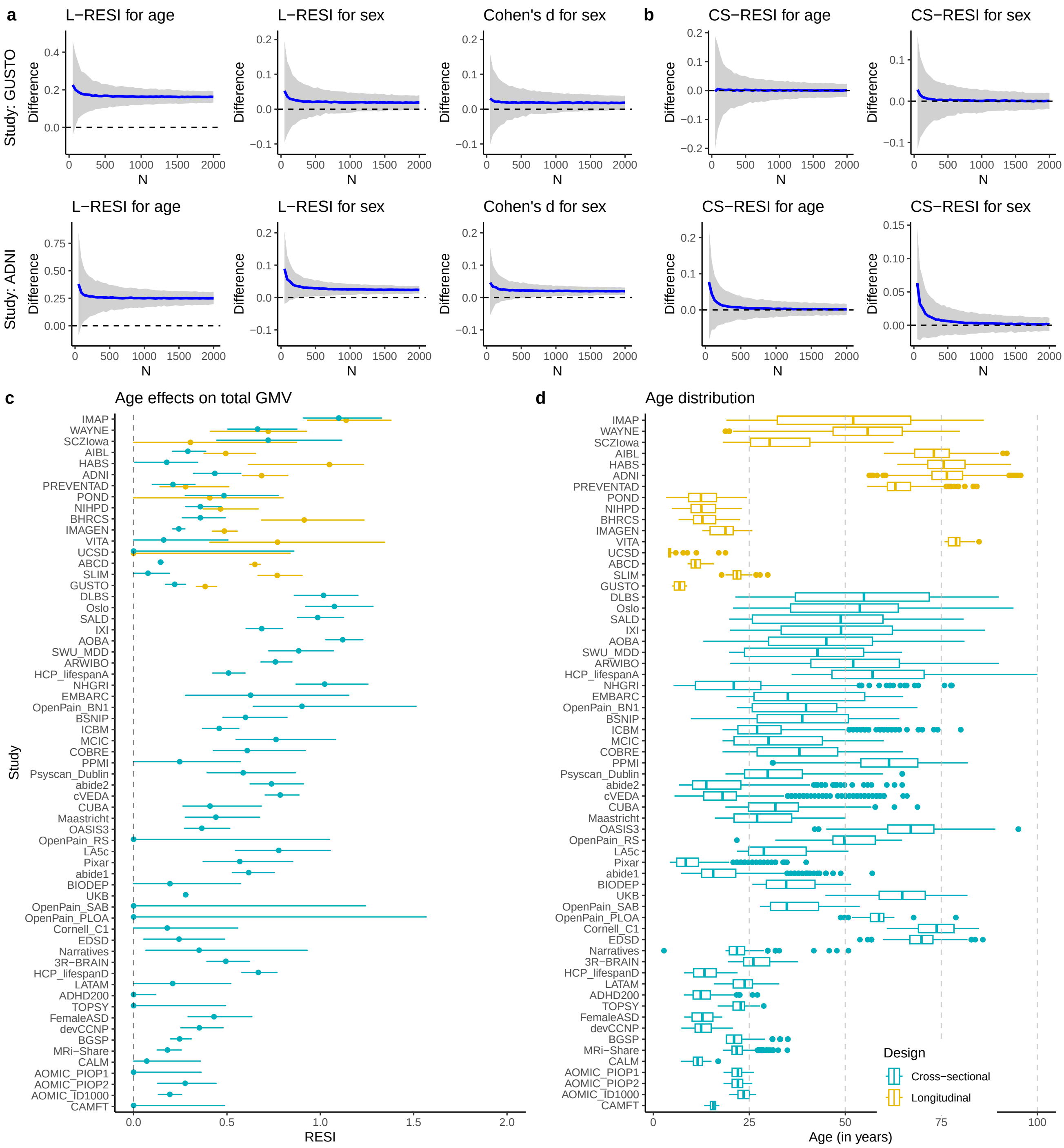

Figure S10: (a) Bootstrap analysis shows that standardized effect sizes (ESs) of age and sex on total gray matter volume (GMV) in longitudinal studies are systematically larger than the standardized ESs-effect sizes in cross-sectional studies (Difference = L-RESI - RESI) and (b) the CS-RESI for longitudinal analysis estimates and removes the difference (Difference = CS-RESI - RESI). We conduct 1,000 bootstraps per sample size (indicated by N on the x-axes). The blue curves represent the mean difference curves and the gray areas are the 95% confidence bands from the bootstraps. (c) Forest plot of the standardized ESs for total GMV-age association shows that the CS-RESIs in longitudinal studies are more comparable to the regular RESIs in cross-sectional studies. Regular RESIs (blue) are estimated for cross-sectional studies, CS-RESIs (blue) and L-RESIs (yellow) are both estimated for longitudinal studies. The dots and lines represent RESI estimates and their 95% confidence intervals (CIs). The studies are sorted by study design and decreasing standard deviation (SD) of age in each study. (d) The age distribution in each study; studies with smaller SD of age tend to have smaller age effects.

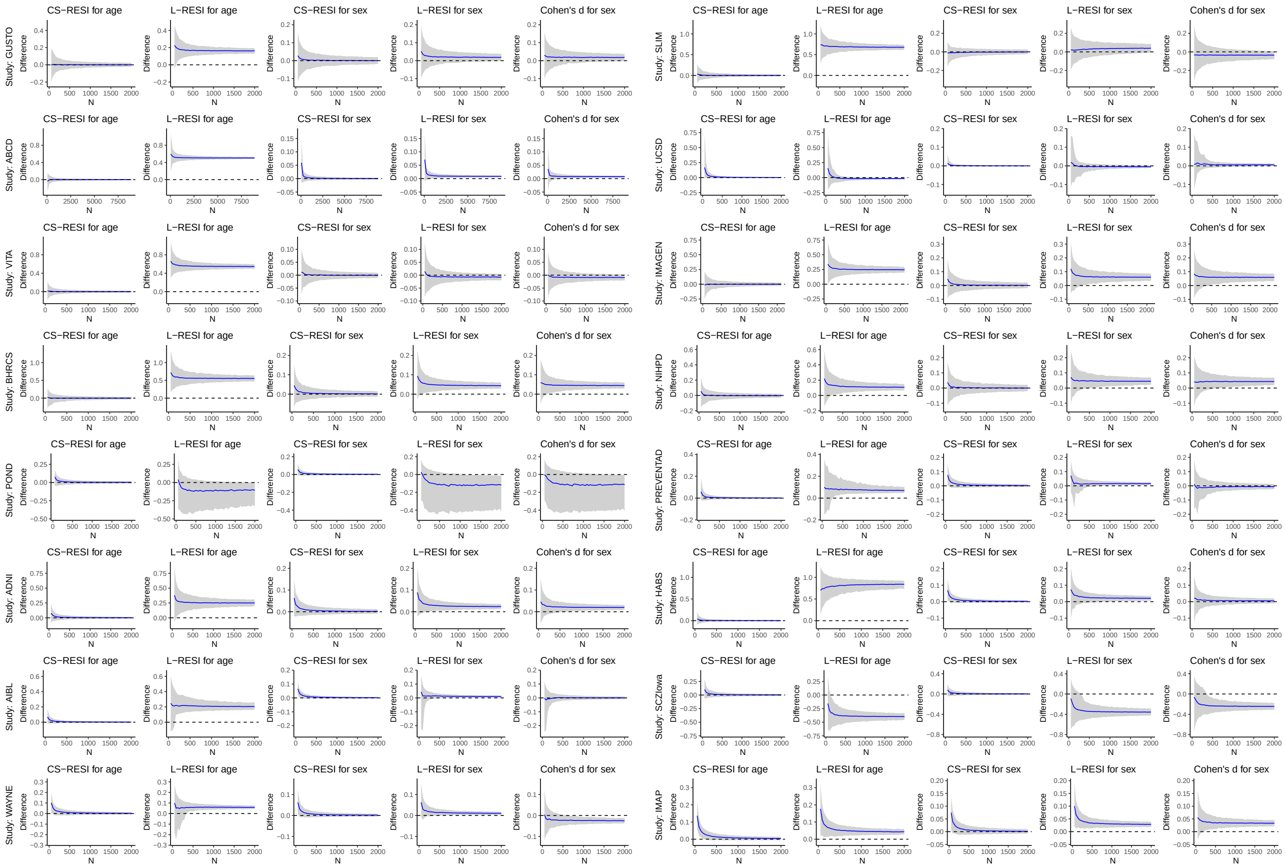

Figure S11: The systematic difference in standardized effect size (ES) by study designs and the comparability of CS-RESI (cross-sectional RESI) in longitudinal studies to RESI in cross-sectional studies. We conduct 1,000 bootstraps per sample size N. The blue curves represent the mean difference curves and the gray areas are the 95% confidence bands from the bootstraps. For most of the studies, the differences of L-RESI (longitudinal RESI) in longitudinal datasets to regular RESI in cross-sectional datasets approach non-zero constants as sample size increases. However, the differences of CS-RESI in longitudinal datasets to the regular RESI in cross-sectional datasets always approach zero at large sample sizes, indicating the systematic difference in standardized ESseffect sizes between longitudinal and cross-sectional datasets is removed by using CS-RESI.

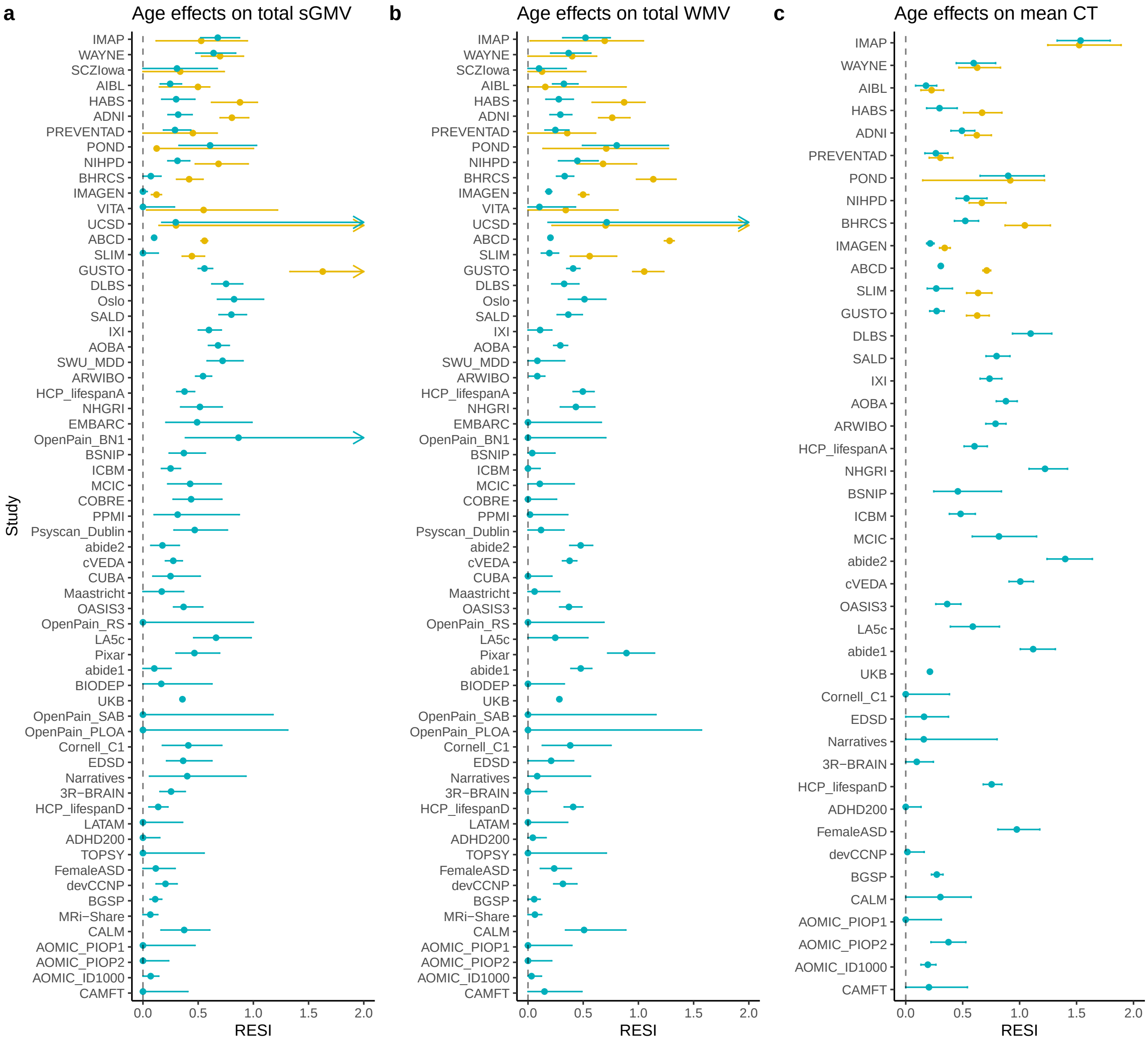

Figure S12: Forest plots of age effects on total subcortical gray matter volume (sGMV), total white matter volume (WMV) and mean cortical thickness (CT), respectively. The age effects on total sGMV and WMV are estimated in 63 studies and the age effects on mean CT are estimated in 43 studies where mean CT is available. RESIs (blue) are estimated for cross-sectional studies, both CS-RESIs (blue) and L-RESIs (yellow) are estimated for longitudinal studies. The dots and lines represent the point estimates of the RESIs and their 95% confidence intervals (CIs). The blue dots and lines represent the CS-RESIs in longitudinal and RESIs in cross-sectional studies. The yellow dots and lines are for the L-RESIs in longitudinal studies. Arrows mean the upper limits of the CIs are greater than the axis boundary.

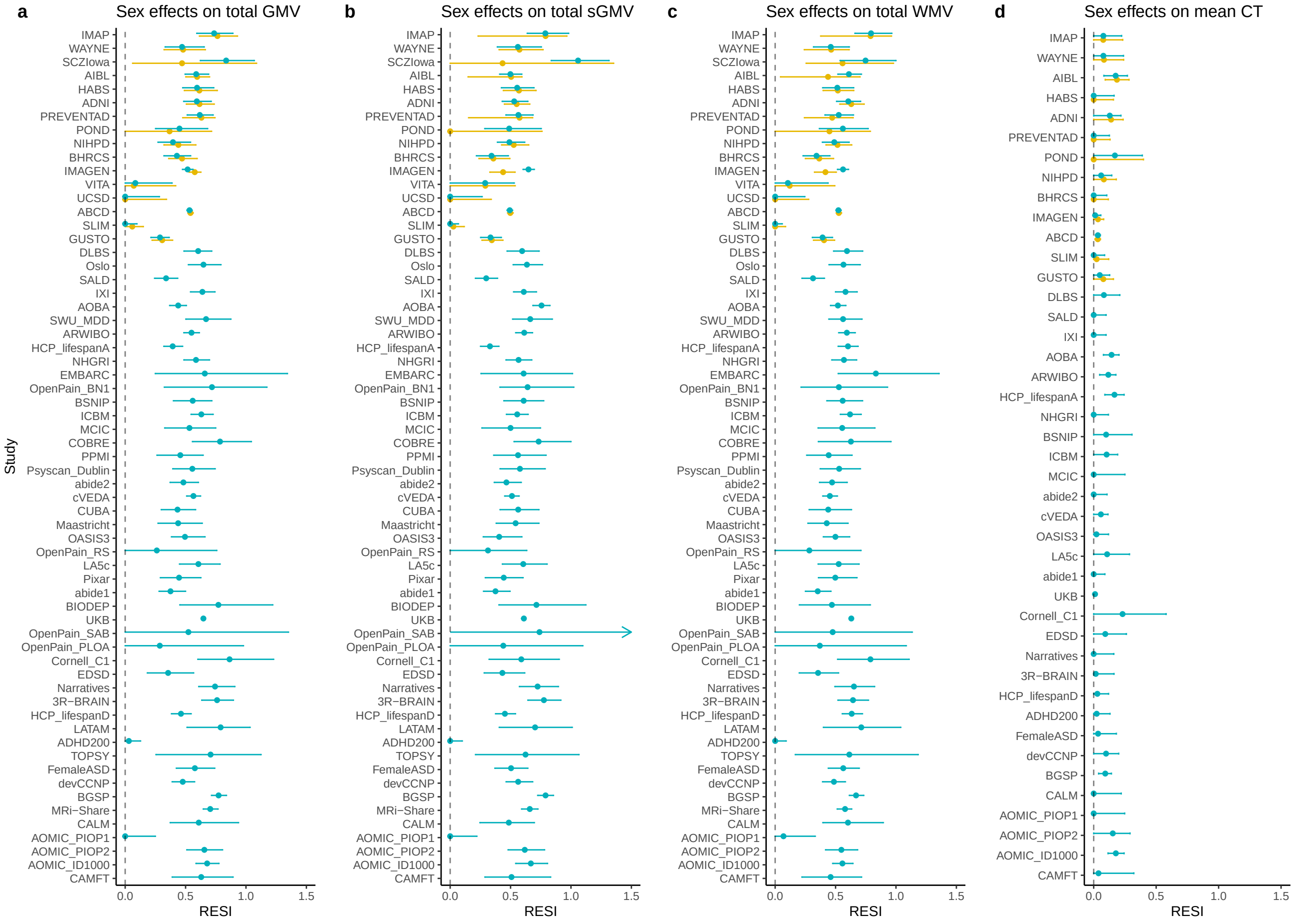

Figure S13: Forest plots of sex effects on total gray matter volume (GMV), total subcortical gray matter volume (sGMV), total white matter volume (WMV) and mean cortical thickness (CT), respectively. The sex effects on total GMV, sGMV and WMV are estimated in 63 studies and the sex effects on mean CT are estimated in 43 studies where mean CT is available. Both CS-RESI (blue) and L-RESI (yellow) are estimated for longitudinal studies. The dots and lines represent the point estimates of the RESIs and their 95% confidence intervals (CIs). The blue dots and lines represent the CS-RESIs in longitudinal and RESIs in cross-sectional studies. The yellow dots and lines are for the L-RESIs in longitudinal studies. Arrow means the upper limit of the CI is greater than the axis boundary.

4 Modified Sampling Design Considerations

4.1 Sampling estimates for targeted sampling

As we show in the main paper, we can use modified sampling designs to obtain a sample with a particular histogram shape to increase covariate variance, standardized ESs and replicability. The decision to pursue this design can be determined by the choices in Extended Data Fig. 7-8. An estimate of the additional number of participants needed to reach a particular sample size using the modified design can be computed if the population distribution of the covariate and modified sampling distribution are known. We assume that we can decide whether to scan each participant, whose covariate values are collected first. To compute the expected number of participants needed to collect, we assume that the data are binned on a consistent grid such as that used to make a histogram of the empirical data and that, to obtain a sample from the target modified distribution g, we will randomly sample from the true distribution f. In this case, both f and g can be treated as multinomial distributions. Assume that each distribution has K bins on the same grid, then the probability the total sample size,

N is larger than m, when trying to obtain a sample of size n from the modified distribution is

$$\mathbb{P}(N > m) = \mathbb{P}(N_k < n\pi_{gk} \text{ for one } k = 1, \dots, K) \tag{9}$$
$$= 1 - \mathbb{P}(N_k \geq n\pi_{gk} \text{ for all } k = 1, \dots, K) \tag{10}$$

where $(N_1, \dots, N_K) \sim \text{multinom}(\pi_{f1}, \dots, \pi_{fK}, m)$ are multinomial. Summing this over all possible values of m gives the expected total sample size, N, required to obtain n samples that exactly have the target distribution using the integrated tail probability formula (see e.g., [13]). Code to evaluate this probability is available in Section 6.

Table S18: Required total sample size, N, when true distribution of covariate is a normal distribution in order to obtain a sample size of n from a uniform distribution for the covariate.

n	N
50	298
100	516
250	1155
500	2201

Besides optimizing study efficiency, there are non-statistical factors that should be considered when choosing a study design, such as the practical challenges in recruiting the study population. Because of the complexity of resources at different study sites, it is hard to provide specific details of how to execute the modified sampling without information about the covariate and how the imaging data are collected. For example, if the covariate varies slowly over time, the measurement can be ascertained in an initial screening and then a subset can be selected for imaging on a later date based on their measured values. If the covariate varies with cognitive state, then it might be necessary to administer cognitive testing and immediately scan a subset or to schedule the imaging and re-administer the cognitive test at scanning. Such complex design issues should be determined on a case-by-case basis.

4.2 Implications for multivariate BWAS

It is important to distinguish between univariate and multivariate BWAS. In univariate BWAS, investigators treat the brain measure as the outcome and model these measurements with a non-brain covariate. In contrast, multivariate BWAS treat the non-brain measurement as the outcome and use machine learning methods to predict the outcome variable as a function of the imaging variable. Multivariate BWAS tend to have larger standardized ESs than univariate BWAS because the signal in neuroimaging data is likely to be dispersed across networks of brain regions. Our analyses focus on univariate BWAS analyses with tens to thousands of brain measurements; our findings directly generalize to higher-dimensional mass-univariate analyses because the standardized ESs do not depend on the number of comparisons although can be biased after selecting regions using multiplicity adjustment. Our findings may generalize to the multivariate BWAS setting, where the imaging variable is used as the covariate, based on previous research that optimizes power using outcome-dependent sampling and two-phase study designs, but further research is merited.

5 Longitudinal Design and Analysis Considerations

Two factors affect standardized ESs such as equation (3): the value of the target parameter (determined through the term involving β) and the efficiency of the estimator of the target parameter (determined by Σ_β). In the main paper, we discuss how between- and within-subject effects may differ across brain non-brain association pairs. When this is true, but the longitudinal model falsely assumes they are equal, then the expected value of the estimated parameter is a weighted average of the two.

To understand how the expected value of the parameter estimate is affected, we assume the true model

$$\begin{aligned} Y_{ij} &= \beta_0 + \beta_b \bar{x}_i + \beta_w (x_{ij} - \bar{x}_i) + \epsilon_{ij} \\ \mathrm{Var}(\epsilon_{ij}) &= \Sigma_\epsilon, \end{aligned} \tag{11}$$

where we treat Σ_ϵ as a known exchangeable covariance matrix. $i = 1, \ldots, n$ indicates the participant and $j = 1, \ldots, m$ indicates the number of measurements, which is assumed to be the same across all participants. This model can be fit using LMMs and GEEs. β_b represents the between-subject effects and β_w represents the within-subject effects. See [14] for the distinction of random effects and between- and within-subject effects.

Unless explicitly parameterized, as in (11), the fitted model for longitudinal data in BWAS often assumes a mean model with $\beta_b = \beta_w$,

$$Y_{ij} = \beta_0 + \beta x_{ij} + \epsilon_{ij}. \tag{12}$$

This is the most common type of model specification used in BWAS. We hypothesized in the main paper that the detrimental effects of increasing within-subject variance or using a longitudinal study were due to the use of model (12) when model (11) was correct. When incorrectly specified with model (12), the expected value of the estimator for β is a combination of the underlying true between- and within-subject effects given by

$$\begin{aligned} \mathbb{E}\hat{\beta} = \beta_b &\left[\frac{(1-\rho)\rho_x^2}{(1+(m-1)\rho)(1-\rho_x^2) + (1-\rho)\rho_x^2} \right] \\ + \beta_w &\left[\frac{(1+(m-1)\rho)(1-\rho_x^2)}{(1+(m-1)\rho)(1-\rho_x^2) + (1-\rho)\rho_x^2} \right] \end{aligned} \tag{13}$$

where $\rho_x^2 = \frac{\sigma_b^2}{\sigma^2}$, which represents the within-subject correlation strength of the covariate x_{ij}, the σ_b^2 and σ^2 denote the between-subject and total variance of the covariate, and ρ is the correlation of the errors or outcome ϵ_{ij}. Derivation of this value and subsequent values are given in Section 5.4. Assuming the variance matrix Σ_ϵ is known, the variance of $\sqrt{n}\hat{\beta}$ is

$$\mathrm{Var}(\sqrt{n}\hat{\beta}) = \left[m \frac{(1+(m-1)\rho)\sigma_w^2 + (1-\rho)\sigma_b^2}{\sigma_\epsilon^2(1+(m-1)\rho)(1-\rho)} \right]^{-1},$$

where σ_w^2 is the within-subject variance of the covariate x_{ij}, σ_ϵ^2 is the error variance, and all other objects are as defined above. The signed RESI ES (4) is a function of the expected value of $\hat{\beta}$ and its variance.

$$\begin{aligned} S_{\mathrm{sgn}} = \frac{\mathbb{E}\hat{\beta}}{\sqrt{\mathrm{Var}(\sqrt{n}\hat{\beta})}} &= m^{1/2}\{(1+(m-1)\rho)\sigma_w^2 + (1-\rho)\sigma_b^2\}^{-1/2}\sigma_y^{-1/2} \\ &\times \left\{ \beta_b \sigma_b^2 \left[\frac{(1-\rho)}{1+(m-1)\rho} \right]^{1/2} + \beta_w \sigma_w^2 \left[\frac{(1+(m-1)\rho)}{(1-\rho)} \right]^{1/2} \right\}. \end{aligned} \tag{14}$$

Equation (13) implies that, when using a single parameter to model the between- and within-subject effects, modifications of the longitudinal study design can change the target parameter, as well as the estimation efficiency, so can affect the numerator and denominator of the ES in equation (14). The formula is visualized in Figure S14. The expected value of the parameter estimator is a convex combination of the between- and within-subject parameters. The expected value of the ES estimator is a linear combination of β_b and β_w, where the weights are complicated functions of the within-subject correlation of the outcome, the number of measurements, and the between- and within-subject variance of the covariate. As shown in the main paper (Fig. 6e-f), the between- and within-subject effects β_b and β_w can differ. In the paper, because our sampling schemes control the variance at the first timepoint and the relative squared distance from the first timepoint, the parameters we estimate are those given in equation (16).

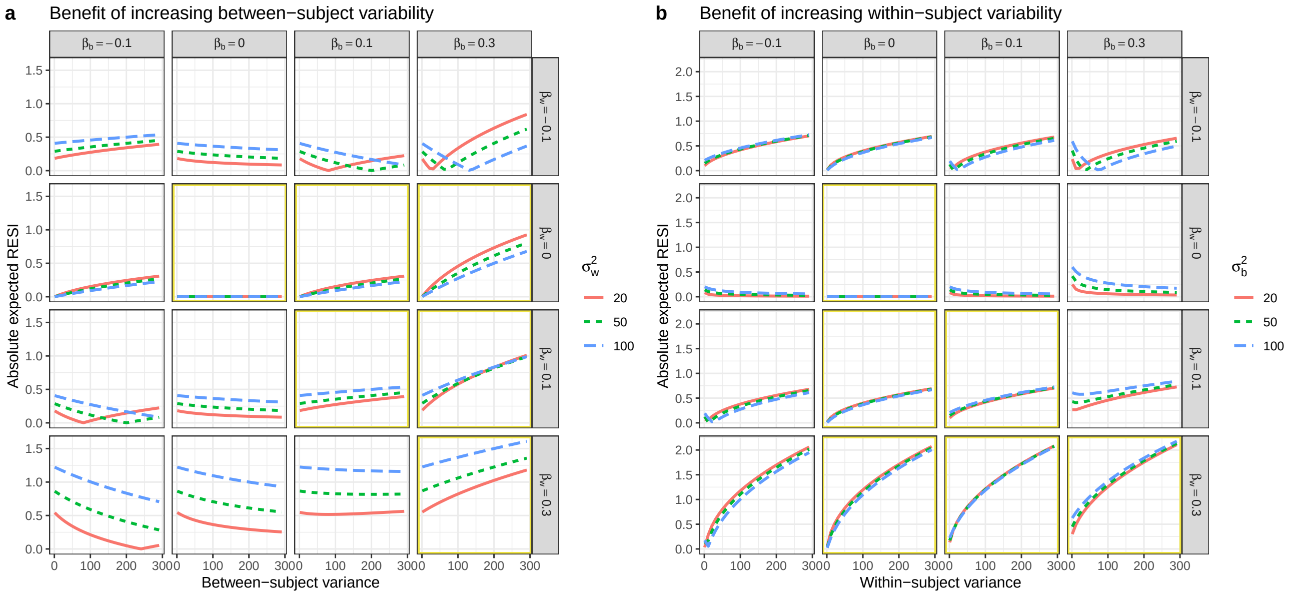

Figure S14: The expected ESs for β in model (12) assuming an exchangeable correlation structure. (a) The benefit of increasing between-subject variance is dependent on the between-subject parameter being larger and the same sign as the within-subject parameter (i.e., β_w; highlighted in yellow). (b) The benefit of increasing within-subject variance is dependent on the within-subject parameter being larger and the same sign as the between-subject parameter (i.e., β_b; highlighted in yellow). When equal ($\beta_b = \beta_w$; diagonal), there is a benefit to increasing either. Note, these changes to the ES are related to bias in the estimation of the parameter due to differing between- and within-subject effects. In all other cases, the curves are negative or nonmonotonic. The formula for the curves is given in (14) and shown for $y^2 = 30$ and $\rho = 0.6$. Absolute expected ESs are shown here as their directions do not affect power.

When fitting a cross-sectional version of model (12) to a randomly selected observation from each participant, as in (6), the expected value of the parameter is

$$\mathbb{E}(\hat{\beta}_{CS}) = \beta_b \rho_x^2 + \beta_w(1 - \rho_x^2).$$

This is derived in Section 5.4, but is also obtained by setting $\rho = 0$ and $m = 1$ in (13). These results imply that the longitudinal model (12) is estimating a different parameter than an equivalent cross-sectional model because it incorporates the correlation of the outcome into the weighting. Changing features of a longitudinal design change the target parameter of (12), which will also affect the standardized ES. In most cases, if the between- and within-subject associations are different, they should be estimated with separate parameters. We propose three different strategies that can be used to model the association between the brain measure and non-brain covariate in BWAS. The first assumes that the between- and within-subject effects are equal and the second and third assume they are different.

5.1 Equal between- and within-subject effects

When $\beta_b = \beta_w = \beta$ then there is no model misspecification and increasing the between- or within-subject variance of the covariate increases ESs for the parameter β. In this case, the parameters β_b and β_w can be distributed out of the sum in (14) and the whole ES increases linearly with respect σ_w and σ_b.

When designing a study to estimate β, it is useful to know which study design is more efficient per participant and per measurement (such as per MRI scan). To do this we compare the ratio of the variances of the estimator obtained from a cross-sectional design $\hat{\beta}_{CS}$, to that obtained in a longitudinal design $\hat{\beta}_L$, while assuming the total number of scans in each study is the same, i.e. $n = Nm$.

$$\begin{aligned}\frac{\text{var}(\hat{\beta}_{\text{CS}})}{\text{var}(\hat{\beta}_{\text{L}})} &= \left[(Nm)\frac{(1+(m-1)\rho)\sigma_w^2 + (1-\rho)\sigma_b^2}{\sigma_\epsilon^2(1+(m-1)\rho)(1-\rho)}\right] \times \frac{\sigma_\epsilon^2}{n\sigma^2} \\ &= \frac{(1+(m-1)\rho)(1-\rho_x^2) + (1-\rho)\rho_x^2}{(1+(m-1)\rho)(1-\rho)}. \end{aligned} \tag{15}$$

Setting this ratio greater than one and solving for ρ, we have

$$\rho > \frac{(m\rho_x^2 - 1)}{(m-1)},$$

which indicates that if the within-subject correlation of the outcome conditional on the covariate is large (i.e., if the within-subject error of brain measure is low) relative to m times the within-subject correlation of the covariate, then the longitudinal design will be more efficient.

Here we provide some example code of how to estimate the within-subject correlation of the outcome (i.e., the parameter ρ above) and the within-subject correlation of the covariate (i.e., the parameter ρ_x^2 above) in GEEs using the R package `geeglm`. We regress the age and sex effects on total GMV in ADNI data for illustration, and we only restrict to the first and last visits of the ADNI participants (i.e., to set $m = 2$). In the following code, the object `ADNI` is a data frame containing all the variables needed, with the continuous variable age (in years) and binary variable sex under the variable names `age_years` and `sex_01`, respectively. The outcome variable `GMV_10000_combat` is the total GMV after removing the site effect using longComBat[15] and scaled by 10,000.

```
# Fit the GEE model with an exchangeable correlation structure
library(geepack)
fit <- geeglm(GMV_10000_combat ~ sex_01 + age_years,
              data = ADNI, corstr = "exchangeable",
              id = participant)
summary(fit)
```

From the output produced by `summary(fit)` below, we can find the estimate for the parameter within-subject correlation of the outcome to be 0.902 by looking at the estimate of `alpha` under `Estimated Correlation Parameters`.

```
Call:
geeglm(formula = GMV_10000_combat ~ sex_01 + age_years, data = ADNI,
    id = participant, corstr = "exchangeable")

 Coefficients:
            Estimate Std.err Wald Pr(>|W|)
(Intercept)  58.6207  1.1957 2404   <2e-16 ***
sex_01        3.7981  0.3030  157   <2e-16 ***
age_years    -0.2574  0.0156  272   <2e-16 ***
---
Signif. codes:  0 '***' 0.001 '**' 0.01 '*' 0.05 '.' 0.1 ' ' 1

Correlation structure = exchangeable
Estimated Scale Parameters:

            Estimate Std.err
(Intercept)     9.93   0.705
  Link = identity

Estimated Correlation Parameters:
      Estimate Std.err
alpha    0.902  0.0101
Number of clusters:   417  Maximum cluster size: 2
```

The within-subject correlation of covariate age can be estimated in R as below:

```
library(magrittr, dplyr)
# Calculate the within-subject correlation of covariate age
## 1. calcualte the between-subject variance of age
sigma2_b = ADNI %>% group_by(participant) %>%
    summarise(subj_mean_age = mean(age_years)) %>%
    ungroup() %>% summarise(sigma2_b = var(subj_mean_age)) %>%
    as.numeric
## 2. calculate the total variance of age
sigma2 = var(ADNI$age_years)
## the within-subject correlation of age
rho2_x = sigma2_b / sigma2
rho2_x
[1] 0.878
```

The estimated within-subject correlations of the outcome and the covariate are both pretty high, with $\hat{\rho} = 0.902$ and $\hat{\rho}_x^2 = 0.878$. Plugging them into the equation (15) along with $m = 2$ shows that the estimated ratio of efficiency is 1.71, indicating that, given a fixed total number of scans n, scanning $n/2$ participants twice and doing a longitudinal analysis is 71% more efficient than doing a cross-sectional analysis with scanning each of n participants once when studying the total GMV-age association in situations of this study.

Differentiating (15) with respect to m shows how the efficiency changes with respect to changes in the number of scans per participant, keeping the number of total scans fixed

$$\frac{d}{dm}\frac{\mathrm{var}(\hat{\beta}_{\mathrm{CS}})}{\mathrm{var}(\hat{\beta}_{\mathrm{L}})} = \frac{-\rho\rho_x^2}{(1+(m-1)\rho)^2}.$$

This function is non-positive for all values of $m > 1$, indicating a reduction in efficiency relative to the cross-sectional design with increasing number of measurements per participant, if the within-subject correlation of the outcome and the within-subject correlation of covariate are both fixed with

increasing m. These formulas explain the results in Fig. 4d, where adding one additional measurement is beneficial, but more than one additional measurement has a diminishing benefit.

In this setting and if the data are cross-sectional, a model such as

$$Y_i = \beta_0 + \beta x_i + \epsilon_i,$$

is appropriate. If the data are longitudinal, a linear mixed model (LMM) or generalized estimating equation (GEE) as in (12) is appropriate.

5.2 Modeling different between- and within-subject effects

When the between- and within-subject effects are different it is probably best to model them separately because it avoids averaging the larger effect with the smaller effect, and because the distinction between these two effects can inform our understanding of brain-behavior associations. In this case, increasing between- or within-subject variance independently increases the ES for the between- or within-subject parameter when (11) is the correct model. To fit this model, we include the original covariate x_{ij} which varies over measurements from the same individual, and also include a covariate that is the mean of the original covariate $\bar{x}_i = n_i^{-1} \sum_{j=1}^{n_i} x_{ij}$, as in equation (11). The mean covariate will be the same value for all measurements within participants. An LMM or GEE can be used to model and estimate the parameters separately. The parameters can be interpreted separately as trait versus state associations with the outcome.

Various parameterizations are possible. They look similar but differ in their interpretation and mathematical formulation. For example, in the main paper, we use an alternative to (11) that models a separate effect for the baseline measurements, which influences all future measurements.

$$Y_{ij} = \beta_0^* + \beta_b^* x_{i1} + \beta_w^* (x_{ij} - x_{i1}) + \epsilon_{ij}. \tag{16}$$

Here, the between- and within-subject variance are defined as

$$\begin{aligned} \sigma_b^2 &= \frac{1}{N} \sum_{i=1}^{N} (x_{i1} - \bar{x}_1)^2 \\ \sigma_w^2 &= \frac{1}{N} \sum_{i=1}^{N} \frac{1}{n_i} \sum_{j=1}^{n_i} (x_{ij} - x_{i1})^2 \end{aligned} \tag{17}$$

This model makes sense if the first measurement in the study represents a particular epoch that is comparable across participants. In the paper, we used this formulation, because our sampling schemes are based on the first measurement and the change from first measurement, which separately affect the between- and within-subject variance shown in (17).

Here, as an example, we provide some example code for estimating the between- and within-subject effects of CBCL on the outcome total GMV (after controlling for age and sex) separately in the ABCD data, as the model specified in (16). First, we need to create variables indicating the subject-specific baseline covariate measures and the change of the covariate from the baselines.

```
# create a variable `x_bl` to indicate
# the subject-specific baseline CBCL values (i.e., x_i1)
ABCD %<>% group_by(temp_id) %>%
    mutate(x_bl = CBCL[eventname == "Baseline"]) %>% ungroup()
# create a variable `x_change` to indicate (x_ij - x_i1)
ABCD %<>% mutate(x_change = CBCL - x_bl)
```

The variables `x_bl` and `x_change` in the data frame object `ABCD` should look like the following:

```
   temp_id eventname        CBCL         x_bl x_change  ...
 1       1 Baseline         36             36  0
 2       2 Baseline         32             32  0
```

```
 3       3 Baseline         51            51  0
 4       4 Baseline         49            49  0
 5       5 Baseline         58            58  0
 6       5 2-year follow-up 61            58  3
 7       5 4-year follow-up 55            58 -3
 8       6 Baseline         51            51  0
 9       6 2-year follow-up 55            51  4
10       6 4-year follow-up 51            51  0
...
```

Then we can estimate the between- and within-subject effects of CBCL in a GEE:

```
# fit a GEE model
library(geeglm)
fit = geeglm(GMV_10000_combat ~ age_years + sex_01 + x_bl + x_change,
              id = temp_id, corstr = "exchangeable", data = ABCD)
summary(fit)

Call:
geeglm(formula = GMV_10000_combat ~ age_years + sex_01 + x_bl +
    x_change, data = ABCD, id = temp_id, corstr = "exchangeable")

 Coefficients:
            Estimate  Std.err     Wald Pr(>|W|)
(Intercept) 77.82355  0.25265 94884.60   <2e-16 ***
age_years   -0.61448  0.01004  3742.46   <2e-16 ***
sex_01       6.04305  0.10527  3295.50   <2e-16 ***
x_bl        -0.04259  0.00481    78.24   <2e-16 ***
x_change    -0.00766  0.00280     7.51   0.0061 **
---
Signif. codes:  0 '***' 0.001 '**' 0.01 '*' 0.05 '.' 0.1 ' ' 1

Correlation structure = exchangeable
Estimated Scale Parameters:
            Estimate Std.err
(Intercept)     33.2    0.47
  Link = identity

Estimated Correlation Parameters:
      Estimate Std.err
alpha    0.915  0.0114
Number of clusters:   11718  Maximum cluster size: 3
```

In the output, the estimates for the between-subject effect and the within-subject effect (i.e., β_b^* and β_w^* in (16)) are the coefficient estimates for the terms `x_bl` and `x_change`.

5.2.1 Interpretation of between- and within-subject effects

The interpretation of the between- and within-subject parameters will be dependent on the context and the parameterization. The parameters can be better understood by looking at the expected differences between two measurements within (18) and between (19) participants,

$$\mathbb{E}(Y_{ij} - Y_{ik}) = \beta_w(x_{ij} - x_{ik}) \tag{18}$$

$$\mathbb{E}(Y_{ij} - Y_{\ell k}) = \beta_b(\bar{x}_i - \bar{x}_k) + \beta_w \left\{(x_{ij} - \bar{x}_i) - (x_{\ell k} - \bar{x}_\ell)\right\}. \tag{19}$$

Comparing measurements for two values of the covariate within a participant is expected to have a different effect than comparing the same two covariate values between participants. From (18), we can see that if we plot $(Y_{ij} - Y_{ik})$ versus $(x_{ij} - x_{ik})$, the slope captures only the within-subject effect, which can be used for visually evaluating the within-subject effect (see e.g., Fig. S12 in the main paper). This can occur if the covariate has high measurement variability, but the outcome variable does not, then we would expect a within-subject change in the covariate to be unrelated to within-subject changes in the outcome (i.e., $\beta_w = 0$). For psychometric measurements that can be affected by the participant's state at measurements, $\bar{x}_i$ in (11) represents participant-specific mean "trait" value of the covariate and the term $(x_{ij} - \bar{x}_i)$ is the within-subject deviation of measurement j from this mean trait value. If the brain outcome measure (e.g., GMV) is expected not to vary by state, then it might be reasonable to believe that $\beta_w \approx 0$, whereas with FC data, it's likely that within-subject association varies across regions as the measurements may be similarly affected by the participant's stat at measurements.

For certain covariates, it does not make sense to consider different between- and within-subject effects, depending on the study design. For example, consider the parameterization in (16) for the GMV-age association in an observational study, it would be odd if the age that a participant entered the study had an additional effect on GMV above the effect of their age when the GMV measurement was taken. In this parameterization, the absence of this first timepoint effect implies $\beta_b^* = 0$, or in the parameterization (11), $\beta_b = \beta_w$. Alternatively, if the study entry represented the occurrence of a particular event, e.g., diagnosis, then it seems plausible that $\beta_b^* \neq 0$.

If there are potential nonlinearities then each effect may be estimated with multiple degrees of freedom, e.g., by using splines. The interpretation of parameter estimates from model (11) will not be comparable to the estimate from a cross-sectional model because the cross-sectional model cannot distinguish the two effects and is estimating the combination. If the goal is to obtain an estimate that is comparable to the one obtained in a cross-sectional study, then see Section 5.3.

5.3 Modeling the averaged parameter

If the true model has different between- and within-subject parameters, despite that it is incorrect, model (12) can still be used. This might be of interest when it is hard to interpret the within-subject effect. In this case, the weighted average from the cross-sectional model depends on whether (11) or (16) is the true model. We recommend targeting the parameter obtained from a cross-sectional model with one measurement per participant (the weighted average of the between- and within-subject parameters),

$$Y_{i1} = \beta_0 + \beta x_{i1} + \epsilon_{i1}. \tag{20}$$

so that longitudinal studies target the same parameter(s) as cross-sectional studies. If (16) is the true model, then that is a special case where the expected value of the cross-sectional model parameter estimator (using model (20)) is equal to the between-subject parameter in Equation (16). When model (20) is fit, but (11) is true, we can use formula (13) to assess the bias of the estimators under different models (Table S19). As can be seen from Table S19, only the GEE with independence working covariance structure targets and unbiasedly estimates the same parameter as the cross-sectional model, but it is still less efficient than a cross-sectional model. The commonly used LMM or GEE with another covariance structure is biased for this parameter, where the bias depends on the structure of the working covariance matrix. In the case of LMM, the working covariance matrix is assumed to be the true covariance. Thus, in this setting, only GEE with independence working covariance can be used to estimate β in (20) in a longitudinal model. In general, when using other correlation structures in GEEs, to increase ES without biasing the parameter estimator, the between- and within-subject variance must be increased proportionally. This is because the weights for the between- and within-subject effects in $\mathbb{E}\hat{\beta}$ are determined by $\rho_x^2 = \frac{\sigma_b^2}{\sigma_b^2 + \sigma_w^2}$ (as defined in Section 5.4); increasing σ_b^2 or σ_w^2 independently will change the value of the parameter. Thus, a sampling scheme that increases both proportionally must be adopted.

The longitudinal model is less efficient per scan than the cross-sectional model under mild assumptions that are likely to hold in BWAS. To see this, we compare the ratio of the variances of the two estimators. Assuming that in each study design we collect Nm total brain measurements, then the ratio

of the variance of the estimator in the longitudinal GEE with independence working covariance to the one of the cross-sectional model (20), is

$$\frac{\mathrm{var}(\hat{\beta}_{\mathrm{GEE}})}{\mathrm{var}(\hat{\beta}_{\mathrm{CS}})} = \frac{\sum_{i=1}^{N}\left(\sum_{j=1}^{m}\sigma_\epsilon^2 X_{ij}^2 + \sum_{j\neq k}^{m}\rho\sigma_\epsilon^2 X_{ij}X_{ik}\right)}{\left(\sum_{i,j}X_{ij}^2\right)^2} \times \frac{\sum_{i}^{Nm}X_{i1}^2}{\sigma_\epsilon^2}$$
$$\geq \frac{\sum_{i,j}X_{ij}^2 + \sum_{i=1}^{N}\sum_{j\neq k}^{m}\rho X_{ij}X_{ik}}{\sum_{i,j}X_{ij}^2} \geq 1,$$

where the first inequality assumes that the behavioral measurements between participants are on average larger than measurements within participants, $\sum_{i}^{Nm}X_{i1}^2 \geq \sum_{i,j}X_{ij}^2$ and the second inequality assumes that brain and behavioral repeated measurements are each positively correlated within-subject. Practically, this analysis indicates that when targeting an averaged parameter (i.e., row 1 of Table S19), it is better to perform a cross-sectional study rather than a longitudinal study. Note, however, that with a fixed number of participants, collecting more longitudinal measurements will still generally increase efficiency relative to a cross-sectional study. Thus, longitudinal data should be used in the GEE framework if it is available.

Table S19: Expected values of the parameter estimates from (20), or (12) under different working covariances. If the goal is to estimate the same parameter as the cross-sectional model (20), then LMM/GEE with non-independence covariance structure are biased, because the weights for β_b and β_w are as given in (13). The only longitudinal model that is unbiased for the cross-sectional effect is the GEE with independence working covariance (second row). $\rho_x^2 = \frac{\sigma_b^2}{\sigma^2}$ as defined in Section 5.4.

Model	Parameters	$\mathbb{E}\hat{\beta}$
Cross-sectional study	$\rho = 0, m = 1$	$\mathbb{E}\hat{\beta} = \beta_b\rho_x + \beta_w(1-\rho_x)$
GEE independence work covariance	$\rho = 0, m > 1$	$\mathbb{E}\hat{\beta} = \beta_b\rho_x + \beta_w(1-\rho_x)$
LMM/GEE exchangeable covariance	$\rho \neq 0, m > 1$	Equation (13)

5.4 Derivation of effect size under exchangeable design

After residualizing x and Y to the intercept design matrix, the expected value of $\hat{\beta}$ is

$$\mathbb{E}(\hat{\beta} \mid \mathbf{x}) = (\mathbf{x}^T\Sigma^{-1}\mathbf{x})^{-1}\mathbf{x}^T\Sigma^{-1}Y = (\mathbf{x}^T\Sigma^{-1}\mathbf{x})^{-1}\mathbf{x}^T\Sigma^{-1}[\bar{\mathbf{x}}, \mathbf{w}]\begin{bmatrix}\beta_b\\ \beta_w\end{bmatrix},$$

where $Y, \mathbf{x}, \bar{\mathbf{x}}, \mathbf{w} \in \mathbb{R}^{nm}$ with

$$\mathbf{x}_{(i-1)\times m+j} = x_{ij} - \bar{x}$$
$$\bar{\mathbf{x}}_{(i-1)\times m+j} = \bar{x}_i - \bar{x}$$
$$\mathbf{w}_{(i-1)\times m+j} = \mathbf{x}_{(i-1)\times m+j} - \bar{\mathbf{x}}_{(i-1)\times m+j}$$
$$\bar{x} = \frac{1}{nm}\sum_{i,j}x_{ij}$$
$$\bar{x}_i = \frac{1}{m}\sum_{j=1}x_{ij}$$

When Σ_ϵ is exchangeable,

$$\Sigma_\epsilon^{-1} = aI + b(I_n \otimes \mathbf{1}\mathbf{1}^T),$$

where $a = (\sigma_\epsilon^2(1-\rho))^{-1}$ and $b = a\left[\frac{-\rho}{(1+(m-1)\rho)}\right]$, $\mathbf{1}$ denotes an m dimensional vector of all ones, I_n is the n dimensional identity matrix, and $\otimes$ denotes the Kronecker product. The inverse variance of $\sqrt{n}\hat{\beta}$

is

$$
\begin{aligned}
\mathrm{Var}(\sqrt{n}\hat{\beta})^{-1} &= n^{-1}(\mathbf{x}^T\Sigma_\epsilon^{-1}\mathbf{x}) \\
&= \frac{m}{\sigma_\epsilon^2(1-\rho)}\left[\sigma^2 - \sigma_b^2\frac{m\rho}{(1+(m-1)\rho)}\right] \\
&= m\left[\frac{(1+(m-1)\rho)\sigma_w^2 + (1-\rho)\sigma_b^2}{\sigma_\epsilon^2(1+(m-1)\rho)(1-\rho)}\right] \\
&= m\frac{\sigma^2}{\sigma_\epsilon^2}\left[\frac{(1+(m-1)\rho)(1-\rho_x^2) + (1-\rho)\rho_x^2}{(1+(m-1)\rho)(1-\rho)}\right] \qquad (21)
\end{aligned}
$$

where

$$
\begin{aligned}
\sigma^2 &= \frac{1}{nm}\sum_{i,j}(x_{ij} - \bar{x})^2 \\
\sigma_b^2 &= \frac{1}{n}\sum_{i=1}^{n}(\bar{x}_i - \bar{x})^2 \\
\sigma_w^2 &= \frac{1}{nm}\sum_{i,j}(x_{ij} - \bar{x}_i)^2 \\
\rho_x^2 &= \frac{\sigma_b^2}{\sigma^2} = \frac{\sigma_b^2}{\sigma_w^2 + \sigma_b^2}.
\end{aligned}
$$

Then $\mathbb{E}\hat{\beta}$ simplifies to

$$
\begin{aligned}
\mathbb{E}\hat{\beta} &= \beta_w + (\beta_b - \beta_w) \times \frac{a\mathbf{x}^T\bar{\mathbf{x}} + b\mathbf{x}^T(I_n \otimes \mathbf{1}\mathbf{1}^T)\bar{\mathbf{x}}}{a\mathbf{x}^T\mathbf{x} + b\mathbf{x}^T(I_n \otimes \mathbf{1}\mathbf{1}^T)\mathbf{x}} \\
&= \beta_w + (\beta_b - \beta_w)m\sigma_b^2(a+bm)\mathrm{Var}(\sqrt{n}\hat{\beta}) \\
&= \beta_w + (\beta_b - \beta_w)m\frac{\sigma_b^2}{\sigma_\epsilon^2(1+(m-1)\rho)}\mathrm{Var}(\sqrt{n}\hat{\beta}) \\
&= \beta_w + (\beta_b - \beta_w)\left[\frac{(1-\rho)\sigma_b^2}{(1+(m-1)\rho)\sigma_w^2 + (1-\rho)\sigma_b^2}\right] \\
&= \beta_b\left[\frac{(1-\rho)\sigma_b^2}{(1+(m-1)\rho)\sigma_w^2 + (1-\rho)\sigma_b^2}\right] \\
&+ \beta_w\left[\frac{(1+(m-1)\rho)\sigma_w^2}{(1+(m-1)\rho)\sigma_w^2 + (1-\rho)\sigma_b^2}\right] \\
&= \beta_b\left[\frac{(1-\rho)\rho_x^2}{(1+(m-1)\rho)(1-\rho_x^2) + (1-\rho)\rho_x^2}\right] \\
&+ \beta_w\left[\frac{(1+(m-1)\rho)(1-\rho_x^2)}{(1+(m-1)\rho)(1-\rho_x^2) + (1-\rho)\rho_x^2}\right]
\end{aligned}
$$

Combining (21) and (13) gives (14).

6 Sample Size Code for Modified Sampling

```
library(pmultinom)
totalN = function(n, nbin=10, alpha = 0.05, eps=0.001,
              g='unif', f='norm'){
  # target number in each bin
  if(tolower(g)=='unif'){
    gbin = rep(1/nbin, nbin) * n
  }
```

```
  # true distribution of the data
  fp = get(paste0('p', f))
  fq = get(paste0('q', f))
  fdomain = c(fq(alpha/2), fq(alpha/2, lower.tail=FALSE))
  fbin = c(-Inf, seq(fdomain[1], fdomain[2], length.out=nbin-1), Inf)
  fbin = diff(fp(fbin))

  # finds max sample size to get the desired precision
  nmax = 500
  tailProb = 1
  while(tailProb>eps){
      tailProb = 1-pmultinom(lower=gbin, size=nmax,
        probs = fbin, method='exact')
      nmax = nmax + 200
    }
# computes multinomial probabilities
  probs = sapply(0:nmax, function(N){pmultinom(lower=gbin, size=N,
    probs = fbin, method='exact')})
  # Using expectation of CDF to compute this expectation
  sum(1-probs)
}
```

7 Consortium Member Information

The members from the consortia used in this manuscript include:

Lifespan Brain Chart Consortium:

Chris Adamson[1,2], Sophie Adler[3], Aaron F. Alexander-Bloch[4,5,6], Evdokia Anagnostou[7,8], Kevin M. Anderson[9], Ariosky Areces-Gonzalez[10,11], Duncan E. Astle[12], Bonnie Auyeung[13,14], Muhammad Ayub[15,16], Jong Bin Bae[17], Gareth Ball[1,18], Simon Baron-Cohen[14,19], Richard Beare[1,2], Saashi A. Bedford[14], Vivek Benegal[20], Richard A.I. Bethlehem[14,21], Frauke Beyer[22], John Blangero[23], Manuel Blesa Cábez[24], James P. Boardman[24], Matthew Borzage[25], Jorge F. Bosch-Bayard[26,27], Niall Bourke[28], Edward T. Bullmore[29], Vince D. Calhoun[30], Mallar M. Chakravarty[31,27], Christina Chen[32], Casey Chertavian[6], Gaël Chetelat[33], Yap S. Chong [34,35], Aiden Corvin[36], Manuela Costantino[37,38], Eric Courchesne[39,40], Fabrice Crivello[41], Vanessa L. Cropley[42], Jennifer Crosbie[43], Nicolas Crossley[44,45], Marion Delarue[33], Richard Delorme[46,47], Sylvane Desrivieres[48], Gabriel Devenyi[49,50], Maria A. Di Biase[42,51], Ray Dolan[52,53,54], Kirsten A. Donald[55,56], Gary Donohoe[57], Lena Dorfschmidt[4,5,6], Katharine Dunlop[58], Anthony D Edwards[59,60,61], Jed T. Elison[62], Cameron T. Ellis[9,63], Jeremy A. Elman[64], Lisa Eyler[65,66], Damien A. Fair[62], Paul C. Fletcher[67,68], Peter Fonagy[69,70], Carol E. Franz[71], Lidice Galan-Garcia[72], Ali Gholipour[73], Jay Giedd[74,75], John H. Gilmore[76], David C. Glahn[77,78], Ian M. Goodyer[29], P. E. Grant[79], Nynke A. Groenewold[80,56], Shreya Gudapati[4,5,6], Faith M. Gunning[81], Raquel E. Gur[4,6], Ruben C. Gur[4,82], Christopher F. Hammill[43,83], Oskar Hansson[84,85], Trey Hedden[86,87], Andreas Heinz[88], Richard N. Henson[12,89], Katja Heuer[90], Jacqueline Hoare[91], Bharath Holla[92,93], Avram J. Holmes[94], Hao Huang[95], Kiho Im[96,97], Jonathan Ipser[98], Clifford R. Jack Jr[99], Andrea P. Jackowski[100,101], Tianye Jia[102,103,104], David T. Jones[105,106], Peter B. Jones[29,107], Rene S Kahn[108,109], Hasse Karlsson[110,111], Linnea Karlsson[110,111], Ryuta Kawashima[112], Elizabeth A. Kelley[113], Silke Kern[114,115], Ki-Woong Kim[116,117,118], Manfred G. Kitzbichler[29], William S. Kremen[64], François Lalonde[119], Brigitte Landeau[33], Jason Lerch[120,121,122], John D. Lewis[123], Jiao Li[124], Wei Liao[124], Conor Liston[125], Michael V. Lombardo[126,14], Jinglei Lv[127,42], Travis T. Mallard[128], Machteld Marcelis[129], Samuel R. Mathias[77], Bernard Mazoyer[41,130], Philip McGuire[131], Michael J. Meaney[132], Andrea Mechelli[131], Bratislav Misic[133], Sarah E Morgan[134,135,136], David Mothersill[137,138,139], Cynthia Ortinau[140], Rik Ossenkoppele[141,142], Minhui Ouyang[95], Lena Palaniyappan[143], Leo Paly[33], Pedro M. Pan[144,145], Christos Pantelis[146,147,148], Min Tae M. Park[149], Tomas Paus[150,151], Zdenka Pausova[43,152], Deirel Paz-Linares[10,153], Alexa Pichet Binette[154,155], Karen Pierce[156], Xing Qian[157], Anqi Qiu[158], Armin Raznahan[119], Timothy Rittman[159], Amanda Rodrigue[77], Caitlin K. Rollins[160,161], Rafael Romero-Garcia[29,162], Lisa Ronan[29], Monica D. Rosenberg[163], David H. Rowitch[164], Giovanni A. Salum[165,166], Theodore D. Satterthwaite[167,4], H. Lina Schaare[168,169], Jenna Schabdach[4,5,6], Russell J. Schachar [43], Michael Schöll[170,171,172], Aaron P. Schultz[173,174,78], Jakob Seidlitz[4,5,6], David Sharp[175,176], Russell T. Shinohara[32,177], Ingmar Skoog[114,115], Christopher D. Smyser[178], Reisa A. Sperling[173,179,78], Dan J. Stein[180], Aleks Stolicyn[181], John Suckling[29,182], Gemma Sullivan[24], Benjamin Thyreau[112], Roberto Toro[90,183], Nicolas Traut[184,185], Kamen A. Tsvetanov[159,186], Nicholas B. Turk-Browne[9,187], Jetro J. Tuulari[188,189,190], Christophe Tzourio[191], Étienne Vachon-Presseau[192,193,194], Mitchell J. Valdes-Sosa[72], Pedro A. Valdes-Sosa[195,196], Sofie L. Valk[197], Therese van Amelsvoort[198], Simon N. Vandekar[199,200], Lana Vasung[201], Petra E Vértes[29,202], Lindsay W. Victoria[81], Sylvia Villeneuve[155,203,154], Arno Villringer[22,204], Jacob W. Vogel[167,4], Konrad Wagstyl [205], Yin-Shan Wang[206,207,208], Simon K. Warfield[73], Varun Warrier[209], Eric Westman[210], Margaret L. Westwater[29], Heather C. Whalley[181,211], Simon R. White[29,212], A. Veronica Witte[204,22,213], Ning Yang[206,207,208], B.T. Thomas Yeo[214,215,216], Hyuk Jin Yun[217], Andrew Zalesky[218], Heather J Zar[80,219], Anna Zettergren[114], Juan H. Zhou[157,220,214], Hisham Ziauddeen[29,221,222], Dabriel Zimmerman[4,5,6], Andre Zugman[223,224,145], Xi-Nian Zuo[206,207,208]

3R-BRAIN

Xi-Nian Zuo[206,207,208]

AIBL
Chris Rowe[225]
*A complete list of AIBL researchers can be found at https://www.aibl.csiro.au

Alzheimer's Disease Neuroimaging Initiative
Clifford R. Jack Jr[99]
*A complete listing of ADNI investigators can be found at: http://adni.loni.usc.edu/wpcontent/uploads/how_to_apply/ADNI_Acknowledgement_List.pdf

Alzheimer's Disease Repository Without Borders Investigators (ARWiBo)
Giovanni B. Frisoni[226,227]
*A complete listing of ARWiBo researchers can be found at: www.arwibo.it/acknowledgement.it

CALM Team
Duncan E. Astle[12]
*More information on CALM team members can be found at: http://calm.mrccbu.cam.ac.uk/team/

CCNP
Yin-Shan Wang[206,207,208], Ning Yang[206,207,208] and Xi-Nian Zuo[206,207,208]

COBRE
Vince D. Calhoun[30]

cVEDA
Bharath Holla[92,93]
*A complete listing of cVEDA researchers can be found at: https://cveda-project.org/people/

Harvard Aging Brain Study
Reisa A. Sperling[173,179,78] and Keith A. Johnson[228, 229, 230, 231]

IMAGEN
Sylvane Desrivieres[48], Andreas Heinz[88], Tianye Jia[102,103,104], and Gunter Schumann[232,233,234]

POND
Evdokia Anagnostou[7,8], Muhammad Ayub[15,16], Jennifer Crosbie[43], Christopher F. Hammill[43,83], Elizabeth A. Kelley[113], Jason Lerch[120,121,122], and Russell J. Schachar [43]

The PREVENT-AD Research Group
Alexa Pichet Binette[154,155], and Sylvia Villeneuve[155,203,154]

[1] Developmental Imaging, Murdoch Children's Research Institute, Melbourne, Victoria, Australia. [2] De-

partment of Medicine, Monash University, Melbourne, Victoria, Australia. [3] UCL Great Ormond Street Institute for Child Health, 30 Guilford St, Holborn, London WC1N 1EH. [4] Department of Psychiatry, University of Pennsylvania, Philadelphia, PA 19104. [5] Department of Child and Adolescent Psychiatry and Behavioral Science, The Children's Hospital of Philadelphia, Philadelphia, PA 19104. [6] Lifespan Brain Institute, The Children's Hospital of Philadelphia, Philadelphia, PA 19104. [7] Department of Pediatrics, University of Toronto. [8] Holland Bloorview Kids Rehabilitation Hospital, Toronto, Canada. [9] Department of Psychology, Yale University, New Haven, CT, USA. [10] The Clinical Hospital of Chengdu Brain Science Institute, MOE Key Lab for NeuroInformation, University of Electronic Science and Technology of China, No. 2006, Xiyuan Ave., West Hi-Tech Zone, Chengdu, 611731, China. [11] University of Pinar del Río "Hermanos Saiz Montes de Oca", Cuba. [12] MRC Cognition and Brain Sciences Unit, University of Cambridge, Cambridge UK. [13] Department of Psychology, School of Philosophy, Psychology and Language Sciences, University of Edinburgh, Edinburgh, United Kingdom. [14] Autism Research Centre, Department of Psychiatry, University of Cambridge, Cambridge, CB2 0SZ, UK. [15] Queen's University, Department of Psychiatry, Centre for Neuroscience Studies, Kingston, Ontario, Canada, email: ma84@queensu.ca. [16] University College London, Mental Health Neuroscience Research Department, Division of Psychiatry, London UK. [17] Department of Neuropsychiatry, Seoul National University Bundang Hospital, Seongnam, Korea. [18] Department of Paediatrics, University of Melbourne, Melbourne, Victoria, Australia. [19] Cambridge Lifetime Asperger Syndrome Service (CLASS), Cambridgeshire and Peterborough NHS Foundation Trust, Cambridge, United Kingdom. [20] Centre for Addiction Medicine, National Institute of Mental Health and Neurosciences (NIMHANS), Bengaluru, India 560029. [21] Brain Mapping Unit, Department of Psychiatry, University of Cambridge, Cambridge, CB2 0SZ, UK. [22] Department of Neurology, Max Planck Institute for Human Cognitive and Brain Sciences, Leipzig, 04103, Germany. [23] Department of Human Genetics, South Texas Diabetes and Obesity Institute, University of Texas Rio Grande Valley. [24] MRC Centre for Reproductive Health, University of Edinburgh, UK. [25] Fetal and Neonatal Institute, Division of Neonatology, Children's Hospital Los Angeles, Department of Pediatrics, Keck School of Medicine, University of Southern California, Los Angeles, California USA. [26] McGill Centre for Integrative Neuroscience, Ludmer Centre for Neuroinformatics and Mental Health, Montreal Neurological Institute. [27] McGill University. [28] Department of Brain Sciences, Imperial College London, London UK & Care Research & Technology Centre, UK Dementia Research Institute. [29] Department of Psychiatry, University of Cambridge, Cambridge, CB2 0SZ, UK. [30] Tri-institutional Center for Translational Research in Neuroimaging and Data Science, Georgia State University, Georgia Institute of Technology, and Emory University, Atlanta, GA, USA. [31] Computational Brain Anatomy (CoBrA) Laboratory, Cerebral Imaging Centre, Douglas Mental Health University Institute. [32] Penn Statistics in Imaging and Visualization Center, Department of Biostatistics, Epidemiology, and Informatics, Perelman School of Medicine, University of Pennsylvania, Philadelphia, PA, USA. [33] Normandie Univ, UNICAEN, INSERM, U1237, PhIND "Physiopathology and Imaging of Neurological Disorders", Institut Blood and Brain @ Caen-Normandie, Cyceron, 14000 Caen, France. [34] Singapore Institute for Clinical Sciences, Agency for Science, Technology and Research, Singapore. [35] Department of Obstetrics and Gynaecology, Yong Loo Lin School of Medicine, National University of Singapore, Singapore. [36] Department of Psychiatry, Trinity College, Dublin, Ireland. [37] Cerebral Imaging Centre, Douglas Mental Health University Institute, Verdun, Canada. [38] Undergraduate program in Neuroscience, McGill University, Montreal, Canada. [39] Department of Neuroscience, University of California, San Diego, San Diego, CA 92093, USA. [40] Autism Center of Excellence, University of California, San Diego, San Diego, CA 92037, USA. [41] Institute of Neurodegenerative Disorders, CNRS UMR5293, CEA, University of Bordeaux. [42] Melbourne Neuropsychiatry Centre, University of Melbourne, Melbourne, Australia. [43] The Hospital for Sick Children, Toronto, Canada. [44] Department of Psychiatry, School of Medicine, Pontificia Universidad Católica de Chile, Diagonal Paraguay 362, Santiago 8330077, Chile. [45] Department of Psychiatry, University of Oxford OX3 7JX. [46] Child and Adolescent Psychiatry Department, Robert Debré University Hospital, AP-HP, F-75019, Paris France. [47] Human Genetics and Cognitive Functions , Institut Pasteur, F-75015, Paris France. [48] Social, Genetic and Developmental Psychiatry Centre, Institute of Psychiatry, Psychology & Neuroscience, King's College London, London, United Kingdom. [49] Cerebral Imaging Centre, Douglas Mental Health University Institute, Montreal, QC, Canada, McGill Department of Psychiatry, Montreal, QC, Canada. [50] Department of Psychiatry, McGill University, Montreal, QC, Canada. [51] Department of Psychiatry, Brigham and Women's Hos-

pital, Harvard Medical School, Boston, Massachusetts, United States. [52] Max Planck UCL Centre for Computational Psychiatry and Ageing Research, University College London, London, UK.. [53] Wellcome Centre for Human Neuroimaging, University College London, London, UK. [54] Wellcome Centre for Human Neuroimaging, 12 Queen Square, London WC1N 3AR. [55] Division of Developmental Paediatrics, Department of Paediatrics and Child Health, Red Cross War Memorial Children's Hospital, Klipfontein Road/Private Bag, Rondebosch, 7700/7701, Cape Town, South Africa. kirsty.donald@uct.ac.za.. [56] Neuroscience Institute, University of Cape Town, Cape Town, South Africa. [57] Center for Neuroimaging, Cognition & Genomics (NICOG), School of Psychology, National University of Ireland Galway, Galway, Ireland. [58] Weil Family Brain and Mind Research Institute, Department of Psychiatry, Weill Cornell Medicine. [59] Centre for the Developing Brain, King's College London, London, UK. [60] Evelina London Children's Hospital. [61] MRC Centre for Neurodevelopmental Disorders, London. [62] Institute of Child Development, Department of Pediatrics, Masonic Institute for the Developing Brain, University of Minnesota, Minneapolis, MN, United States. [63] Haskins Laboratories, New Haven, CT, USA. [64] Department of Psychiatry, Center for Behavior Genetics of Aging, University of California, San Diego, La Jolla, CA. [65] Desert-Pacific Mental Illness Research Education and Clinical Center, VA San Diego Healthcare, San Diego, CA, USA. [66] Department of Psychiatry, University of California San Diego, Los Angeles, CA, USA. [67] Department of Psychiatry, University of Cambridge, and Wellcome Trust MRC Institute of Metabolic Science, Cambridge Biomedical Campus, Cambridge, United Kingdom. [68] Cambridgeshire and Peterborough NHS Foundation Trust. [69] Department of Clinical, Educational and Health Psychology, University College London, London, UK. [70] Anna Freud National Centre for Children and Families, London UK. [71] Department of Psychiatry, Center for Behavior Genetics of Aging, University of California, San Diego, La Jolla, CA 92093. [72] Cuban Center for Neuroscience, La Habana, Cuba. [73] Computational Radiology Laboratory, Boston Children's Hospital, Boston, MA 02115. [74] Department of Child and Adolescent Psychiatry, University of California, San Diego, San Diego, CA 92093, USA. [75] Department of Psychiatry, University of California San Diego, San Diego, CA, USA. [76] Department of Psychiatry, University of North Carolina, Chapel Hill, NC, USA. [77] Department of Psychiatry, Boston Children's Hospital and Harvard Medical School, Boston, MA 02115. [78] Harvard Medical School, Boston, MA 02115. [79] Division of Newborn Medicine and Neuroradiology, Fetal Neonatal Neuroimaging and Developmental Science Center, Boston Children's Hospital, Harvard Medical School, Boston, MA 02115, USA. [80] Department of Paediatrics and Child Health, Red Cross War Memorial Children's Hospital, SA-MRC Unit on Child & Adolescent Health, University of Cape Town, South Africa. [81] Weill Cornell Institute of Geriatric Psychiatry, Department of Psychiatry, Weill Cornell Medicine. [82] Lifespan Brain Institute, The Children's Hospital of Philadelphia, Philadelphia, PA 19105. [83] Mouse Imaging Centre, Toronto, Canada. [84] Clinical Memory Research Unit, Department of Clinical Sciences Malmö, Lund University, Malmö, Sweden. [85] Memory Clinic, Skåne University Hospital, Malmö, Sweden. [86] Department of Neurology, Icahn School of Medicine at Mount Sinai, New York, NY 10029, USA. [87] Athinoula A. Martinos Center for Biomedical Imaging, Department of Radiology, Massachusetts General Hospital, Harvard Medical School, Boston, MA 02129, USA. [88] Department of Psychiatry and Psychotherapy, Charite University Hospital Berlin, Berlin, Germany. [89] Department of Psychiatry, University of Cambridge, Cambridge, UK. [90] Institut Pasteur, Université Paris Cité, Unité de Neuroanatomie Appliquée et Théorique, F-75015 Paris, France. [91] Department of Psychiatry, University of Cape Town, Cape Town, South Africa. [92] Department of Integrative Medicine, NIMHANS, Bengaluru-560029, India. [93] Accelerator Program for Discovery in Brain disorders using Stem cells (ADBS), Department of Psychiatry, NIMHANS, Bengaluru-560029, India. [94] Department of Psychiatry, Brain Health Institute, Rutgers University, Piscataway, NJ, USA. [95] Department of Radiology, Children's Hospital of Philadelphia and University of Pennsylvania, Philadelphia, PA 19104. [96] Division of Newborn Medicine, Fetal Neonatal Neuroimaging and Developmental Science Center, Boston Children's Hospital, Harvard Medical School, Boston, MA 02115, USA. [97] Boston Children's Hospital, Boston, MA 02115. [98] Department of Psychiatry and Mental Health, Clinical Neuroscience Institute, University of Cape Town. [99] Department of Radiology, Mayo Clinic, Rochester, MN 55905, USA. [100] Department of Psychiatry, Universidade Federal de São Paulo. [101] National Institute of Developmental Psychiatry, CNPq. [102] Institute of Science and Technology for Brain-Inspired Intelligence, Fudan University, Shanghai, 200433, China. [103] Key Laboratory of Computational Neuroscience and BrainInspired Intelligence (Fudan University), Ministry of Education, Shanghai, China. [104] Centre for Population Neuroscience

and Precision Medicine (PONS), Institute of Psychiatry, Psychology and Neuroscience, SGDP Centre, King's College London, London SE5 8AF, UK. [105] Department of Neurology, Mayo Clinic, Rochester, MN, USA. [106] Department of Radiology, Mayo Clinic, Rochester, MN, USA. [107] Cambridgeshire and Peterborough NHS Foundation Trust, Huntingdon, United Kingdom. [108] Department of Psychiatry, Icahn School of Medicine at Mount Sinai, New York, NY, USA. [109] Department of Psychiatry, Icahn School of Medicine, Mount Sinai, New York, USA. [110] Department of Clinical Medicine, Department of Psychiatry and Turku Brain and Mind Center, FinnBrain Birth Cohort Study, University of Turku and Turku University Hospital, Turku, Finland. [111] Centre for Population Health Research, Turku University Hospital and University of Turku, Turku, Finland. [112] Institute of Development, Aging and Cancer, Tohoku University, Seiryocho, Aobaku, Sendai 980-8575, Japan. [113] Queen's University, Departments of Psychology and Psychiatry, Centre for Neuroscience Studies, Kingston, Ontario, Canada. [114] Neuropsychiatric Epidemiology Unit, Department of Psychiatry and Neurochemistry, Institute of Neuroscience and Physiology, the Sahlgrenska Academy, Centre for Ageing and Health (AGECAP) at the University of Gothenburg, Sweden. [115] Region Västra Götaland, Sahlgrenska University Hospital, Psychiatry, Cognition and Old Age Psychiatry Clinic, Gothenburg, Sweden. [116] Department of Brain and Cognitive Sciences, Seoul National University College of Natural Sciences, Seoul, Republic of Korea. [117] Department of Neuropsychiatry, Seoul National University Bundang Hospital, Seongnam, Republic of Korea. [118] Department of Psychiatry, Seoul National University College of Medicine, Seoul, Republic of Korea. [119] Section on Developmental Neurogenomics, Human Genetics Branch, National Institute of Mental Health, Bethesda, MD, USA. [120] Department of Medical Biophysics, University of Toronto, Toronto, ON, Canada. [121] Mouse Imaging Centre, The Hospital for Sick Children, Toronto, ON, Canada. [122] Wellcome Centre for Integrative Neuroimaging, FMRIB, Nuffield Department of Clinical Neuroscience, University of Oxford, Oxford, UK. [123] Montreal Neurological Institute, McGill University, Montreal, Canada. [124] The Clinical Hospital of Chengdu Brain Science Institute, University of Electronic Science and Technology of China, Chengdu 611731, China. [125] Department of Psychiatry and Brain and Mind Research Institute, Weill Cornell Medicine. [126] Laboratory for Autism and Neurodevelopmental Disorders, Center for Neuroscience and Cognitive Systems @UniTn, Istituto Italiano di Tecnologia, Rovereto, Italy. [127] School of Biomedical Engineering & Brain and Mind Centre, The University of Sydney, Sydney, NSW, Australia. [128] Department of Psychology, University of Texas, Austin, Texas 78712, USA. [129] Department of Psychiatry and Neuropsychology, School of Mental Health and Neuroscience, EURON, Maastricht University Medical Centre, PO Box 616, 6200 MD, Maastricht, the Netherlands; Institute for Mental Health Care Eindhoven (GGzE), Eindhoven, the Netherlands.. [130] Bordeaux University Hospital. [131] Professor, Department of Psychosis Studies, Institute of Psychiatry, Psychology and Neuroscience, King's College London, UK. [132] Ludmer Centre for Neuroinformatics and Mental Health, Douglas Mental Health University Institute, McGill University, Montreal, Quebec, Canada; Singapore Institute for Clinical Sciences, Singapore. [133] McConnell Brain Imaging Centre, Montreal Neurological Institute, McGill University, Montreal, QC H3A 2B4, Canada. [134] Department of Computer Science and Technology, University of Cambridge, Cambridge CB3 0FD, United Kingdom. [135] Department of Psychiatry, University of Cambridge, Cambridge CB2 0SZ, United Kingdom. [136] The Alan Turing Institute, London, NW1 2DB. [137] Department of Psychology, School of Business, National College of Ireland, Dublin, Ireland. [138] School of Psychology & Center for Neuroimaging and Cognitive Genomics, National University of Ireland Galway, Galway, Ireland. [139] Department of Psychiatry, Trinity College Dublin, Dublin, Ireland. [140] Department of Pediatrics, Washington University in St. Louis, St. Louis, Missouri, United States. [141] Alzheimer Center Amsterdam, Department of Neurology, Amsterdam Neuroscience, Vrije Universiteit Amsterdam, Amsterdam UMC, Amsterdam, The Netherlands. [142] Lund University, Clinical Memory Research Unit, Lund, Sweden. [143] Robarts Research Institute & The Brain and Mind Institute, University of Western Ontario, London, Ontario, Canada. [144] Department of Psychiatry, Federal University of Sao Poalo (UNIFESP). [145] National Institute of Developmental Psychiatry for Children and Adolescents (INPD), Brazil.. [146] Melbourne Neuropsychiatry Centre, Department of Psychiatry, The University of Melbourne and Melbourne Health, Carlton South, Victoria, Australia. [147] Melbourne School of Engineering, The University of Melbourne, Parkville, Victoria, Australia. [148] Florey Institute of Neuroscience and Mental Health, Parkville, VIC, Australia. [149] Department of Psychiatry, Schulich School of Medicine and Dentistry, Western University, London, ON, Canada. [150] Department of Psychiatry, Faculty of Medicine and Centre Hospitalier Universitaire

Sainte-Justine, University of Montreal, Montreal, Quebec, Canada. [151] Departments of Psychiatry and Psychology, University of Toronto, Toronto, ON, Canada.. [152] Departments of Physiology and Nutritional Sciences, University of Toronto, Toronto, Canada. [153] Cuban Neuroscience Center, Havana, Cuba. [154] Department of Psychiatry, Faculty of Medicine, McGill University, Montreal, Qc, H3A 1Y2, Canada. [155] Douglas Mental Health University Institute, Montreal, Qc, H4H 1R3, Canada. [156] Department of Neurosciences, University of California, San Diego La Jolla, CA, USA. [157] Center for Sleep and Cognition, Yong Loo Lin School of Medicine, National University of Singapore, Singapore. [158] Department of Biomedical Engineering, The N.1 Institute for Health, National University of Singapore. [159] Department of Clinical Neurosciences, University of Cambridge, Cambridge UK. [160] Department of Neurology, Harvard Medical School. [161] Department of Neurology, Boston Children's Hospital, Boston, MA 02115. [162] Instituto de Biomedicina de Sevilla (IBiS) HUVR/CSIC/Universidad de Sevilla, Dpto. de Fisiología Médica y Biofísica, Spain. [163] Department of Psychology, Neuroscience Institute, University of Chicago. [164] Department of Paediatrics and Wellcome-MRC Cambridge Stem Cell Institute, University of Cambridge, Hills Road, Cambridge, UK. [165] Department of Psychiatry, Universidade Federal do Rio Grande do Sul (UFRGS). [166] National Institute of Developmental Psychiatry (INPD). [167] Lifespan Informatics & Neuroimaging Center, University of Pennsylvania, Philadelphia, PA 19104. [168] Otto Hahn Group Cognitive Neurogenetics, Max Planck Institute for Human Cognitive and Brain Sciences, Leipzig, Germany. [169] Institute of Neuroscience and Medicine (INM-7: Brain and Behaviour), Research Centre Juelich, Juelich, Germany. [170] Wallenberg Centre for Molecular and Translational Medicine, University of Gothenburg, Gothenburg, Sweden. [171] Department of Psychiatry and Neurochemistry, University of Gothenburg, Sweden. [172] Dementia Research Centre, Queen's Square Institute of Neurology, University College London, UK. [173] Harvard Aging Brain Study, Department of Neurology, Massachusetts General Hospital, Boston, MA 02114. [174] Athinoula A. Martinos Center for Biomedical Imaging, Department of Radiology, Massachusetts General Hospital, Charlestown, MA 02129, USA. [175] Department of Brain Sciences, Imperial College London, London UK . [176] Care Research & Technology Centre, UK Dementia Research Institute. [177] Center for Biomedical Image Computing and Analytics, Department of Radiology, Perelman School of Medicine, University of Pennsylvania, Philadelphia, PA, USA. [178] Departments of Neurology, Pediatrics, and Radiology, Washington University School of Medicine, St. Louis, United States. [179] Center for Alzheimer Research and Treatment, Department of Neurology, Brigham and Women's Hospital, Boston, MA 02115. [180] SA MRC Unit on Risk & Resilience in Mental Disorders, Dept of Psychiatry and Neuroscience Institute, University of Cape Town, Cape Town, South Africa. [181] Division of Psychiatry, Centre for Clinical Brain Sciences, University of Edinburgh, UK. [182] Cambridge and Peterborough Foundation NHS Trust. [183] Université de Paris, Paris, France. [184] Department of Neuroscience, Institut Pasteur, Paris, France. [185] Center for Research and Interdisciplinarity (CRI), Université Paris Descartes, Paris, France. [186] Department of Psychology, University of Cambridge, Cambridge, UK. [187] Wu Tsai Institute, Yale University, New Haven, CT, USA. [188] Department of Clinical Medicine, Department of Psychiatry, FinnBrain Birth Cohort Study, University of Turku, Turku, Finland. [189] Department of Clinical Medicine, University of Turku, Turku Finland. [190] Turku Collegium for Science, Medicine and Technology, University of Turku, Turku, Finland. [191] Univ. Bordeaux, Inserm, Bordeaux Population Health Research Center, U1219, CHU Bordeaux, F-33000 Bordeaux, France. [192] Faculty of Dental Medicine and Oral Health Sciences, McGill University, Montreal, Qc, H3A 1G1, Canada. [193] Faculty of Dentistry, McGill University, Montreal, Qc, H3A 1G1, Canada. [194] Alan Edwards Centre for Research on Pain (AECRP), McGill University, Montreal, Qc, H3A 1G1, Canada. [195] Joint China-Cuba Lab, University of Electronic Science and Technology, Chengdu China/Cuban Center for Neuroscience, La Habana, Cuba. [196] University of Electronic Science and Technology of China/Cuban Center for Neuroscience. [197] Institute for Neuroscience and Medicine 7, Forschungszentrum Juelich; Max Planck Institute for Human Cognitive and Brain Sciences. [198] Department of Psychiatry & Neurosychology, Maastricht University, Maastricht, The Netherlands. [199] Department of Biostatistics, Vanderbilt University, Nashville, Tennessee, USA. [200] Department of Biostatistics, Vanderbilt University Medical Center, Nashville, Tennessee, USA. [201] Division of Newborn Medicine, Fetal Neonatal Neuroimaging and Developmental Science Center, Department of Pediatrics, Boston Children's Hospital, Boston, MA 02115. [202] The Alan Turing Institute, London NW1 2DB, UK. [203] McConnell Brain Imaging Center, Montreal Neurological Institute, McGill University, Montreal, Quebec, Canada. [204] Clinic for Cognitive Neurology, University of Leipzig Medical Center, Leipzig,

04103, Germany. [205] Wellcome Centre for Human Neuroimaging, Institute of Neurology, University College London, WC1N 3AR. [206] State Key Laboratory of Cognitive Neuroscience and Learning, Beijing Normal University, Beijing 100875, China. [207] Developmental Population Neuroscience Research Center, IDG/McGovern Institute for Brain Research, Beijing Normal University, Beijing 100875, China. [208] National Basic Science Data Center, Beijing 100190, China. [209] Department of Psychiatry, University of Cambridge, Cambridge, CB2 0SZ, UK. [210] Division of Clinical Geriatrics, Center for Alzheimer Research, Department of Neurobiology, Care Sciences and Society, Karolinska Institutet, Stockholm, Sweden. [211] Generation Scotland, University of Edinburgh. [212] MRC Biostatistics Unit, University of Cambridge, Cambridge, England. [213] Faculty of Medicine, CRC 1052 'Obesity Mechanisms', University of Leipzig, Leipzig, 04103, Germany. [214] Department of Electrical and Computer Engineering, National University of Singapore, Singapore. [215] Centre for Sleep & Cognition and Centre for Translational MR Research, Yong Loo Lin School of Medicine, National University of Singapore, Singapore. [216] N.1 Institute for Health & Institute for Digital Medicine, National University of Singapore, Singapore. [217] Fetal Neonatal Neuroimaging and Developmental Science Center, Division of Newborn Medicine, Boston Children's Hospital, Harvard Medical School, Boston, MA 02115, USA. [218] Melbourne Neuropsychiatry Centre, University of Melbourne, Melbourne, Australia; Department of Biomedical Engineering, University of Melbourne, Melbourne, Australia.. [219] SAMRC Unit on Child & Adolescent Health, University of Cape Town, South Africa. [220] Center for Translational Magnetic Resonance Research, Yong Loo Lin School of Medicine, National University of Singapore, Singapore. [221] Wellcome Trust-MRC Institute of Metabolic Science, University of Cambridge, Cambridge, CB2 0SZ. [222] Cambridgeshire and Peterborough Foundation Trust, Cambridge, CB21 5EF. [223] National Institute of Mental Health (NIMH), National Institutes of Health (NIH), Bethesda, Maryland, USA. [224] Department of Psychiatry, Escola Paulista de Medicina, São Paulo, Brazil. [225] Memory Disorders Clinic, Austin Health, Melbourne, Victoria, Australia. [226] University Hospitals and University of Geneva, Geneva, Switzerland. [227] IRCCS Fatebenefratelli, The National Centre for Alzheimer's and Mental Diseases, Brescia, Italy. [228] Harvard Medical School, Boston, MA, USA. [229] Harvard Aging Brain Study, Department of Neurology, Massachusetts General Hospital, Boston, MA 02114. [230] Center for Alzheimer Research and Treatment, Department of Neurology, Brigham and Women's Hospital, Boston, MA, USA. [231] Department of Radiology, Massachusetts General Hospital, Boston, MA, USA. [232] Institute of Science and Technology for Brain-Inspired Intelligence, Fudan University, Shanghai, 200433, China. [233] Centre for Population Neuroscience and Precision Medicine (PONS), Institute of Psychiatry, Psychology and Neuroscience, SGDP Centre, King's College London, London SE5 8AF, UK. [234] PONS-Centre, Charite Mental Health, Dept of Psychiatry and Psychotherapy, Charite Campus Mitte, Berlin, Germany.

References

1. Cohen, J. *Statistical power analysis for the behavioral sciences* (Erlbaum Associates, 1988).
2. Chinn, S. A simple method for converting an odds ratio to effect size for use in meta-analysis. *Statistics in Medicine* **19,** 3127–3131 (2000).
3. Morris, S. B. & DeShon, R. P. Combining effect size estimates in meta-analysis with repeated measures and independent-groups designs. *Psychological methods* **7,** 105–125 (2002).
4. Vandekar, S., Tao, R. & Blume, J. A Robust Effect Size Index. *Psychometrika* **85,** 232–246 (2020).
5. Kang, K. *et al.* Accurate Confidence and Bayesian Interval Estimation for Non-centrality Parameters and Effect Size Indices. *Psychometrika* (2023).
6. Hedges, L. V. & Olkin, I. *Statistical Methods for Meta-Analysis* ISBN: 978-0-08-057065-5 (Elsevier, 1985).
7. Rosenthal, R. Parametric measures of effect size. *The handbook of research synthesis* **621,** 231–244 (1994).
8. Zhang, Z. & Schoeps, N. On robust estimation of effect size under semiparametric models. *Psychometrika* **62,** 201–214 (1997).
9. Long, J. S. & Freese, J. *Regression models for categorical dependent variables using Stata* 2nd ed. (Stata press, 2006).
10. Long, J. S. & Ervin, L. H. Using heteroscedasticity consistent standard errors in the linear regression model. *The American Statistician* **54,** 217–224 (2000).
11. Liang, K.-Y. & Zeger, S. L. Longitudinal data analysis using generalized linear models. *Biometrika* **73,** 13–22 (1986).
12. Jones, M., Kang, K. & Vandekar, S. *RESI: An R Package for Robust Effect Sizes* 2023. `http://arxiv.org/abs/2302.12345`.
13. Lo, A. Demystifying the Integrated Tail Probability Expectation Formula. *The American Statistician* **73,** 367–374 (2019).
14. Curran, P. J. & Bauer, D. J. The Disaggregation of Within-Person and Between-Person Effects in Longitudinal Models of Change. *Annual Review of Psychology* **62,** 583–619 (2011).
15. Beer, J. C. *et al.* Longitudinal ComBat: A method for harmonizing longitudinal multi-scanner imaging data. *NeuroImage* **220,** 117129 (2020).
