# Supplementary material for: Study design features increase replicability in brain-wide association studies
Source: Nature. 2024 Nov 27;636(8043):719–27. doi: 10.1038/s41586-024-08260-9 (PMC11655360; doi:10.1038/s41586-024-08260-9)
Supplement: Supplementary file 2 — Reporting Summary [file 41586_2024_8260_MOESM2_ESM.pdf]

nature portfolio

Corresponding author(s): Kaidi Kang; Simon Vandekar

Last updated by author(s): Sep 19, 2024

Reporting Summary

Nature Portfolio wishes to improve the reproducibility of the work that we publish. This form provides structure for consistency and transparency in reporting. For further information on Nature Portfolio policies, see our Editorial Policies and the Editorial Policy Checklist.

Statistics

For all statistical analyses, confirm that the following items are present in the figure legend, table legend, main text, or Methods section.

n/a	Confirmed	
☐	☒	The exact sample size (*n*) for each experimental group/condition, given as a discrete number and unit of measurement
☐	☒	A statement on whether measurements were taken from distinct samples or whether the same sample was measured repeatedly
☐	☒	The statistical test(s) used AND whether they are one- or two-sided<br>*Only common tests should be described solely by name; describe more complex techniques in the Methods section.*
☐	☒	A description of all covariates tested
☐	☒	A description of any assumptions or corrections, such as tests of normality and adjustment for multiple comparisons
☐	☒	A full description of the statistical parameters including central tendency (e.g. means) or other basic estimates (e.g. regression coefficient) AND variation (e.g. standard deviation) or associated estimates of uncertainty (e.g. confidence intervals)
☐	☒	For null hypothesis testing, the test statistic (e.g. *F*, *t*, *r*) with confidence intervals, effect sizes, degrees of freedom and *P* value noted<br>*Give P values as exact values whenever suitable.*
☒	☐	For Bayesian analysis, information on the choice of priors and Markov chain Monte Carlo settings
☐	☒	For hierarchical and complex designs, identification of the appropriate level for tests and full reporting of outcomes
☐	☒	Estimates of effect sizes (e.g. Cohen's *d*, Pearson's *r*), indicating how they were calculated

Our web collection on statistics for biologists contains articles on many of the points above.

Software and code

Policy information about availability of computer code

Data collection	No software was used in the data collection. This study used repository data and did not collect new data.
Data analysis	Data was analyzed by using a combination of open source R code and custom R code which are available on https://github.com/KaidiK/RESI_BWAS. All visualization and statistics represented in graphical format were generated by using the "ggplot2" R package. For multi-site studies, the site effects were removed in advance using the "neuroCombat" and "longCombat" R packages for cross-sectional and longitudinal studies, respectively. In the boxplots shown, they indicate the median and lower and upper hinges correspond to the first and third quartiles (the 25th and 75th percentiles). The upper whisker extends from the hinge to the largest value at most 1.5 * IQR of the hinge. Data beyond the end of the whisker are called "outlier" points and are plotted individually. The linear regression models were fitted using the "lm" function from base "stats" package. The generalized estimating equations (GEEs) were fitted using the "geepack" R package. The robust effect size index estimates and the confidence intervals were derived using the "RESI" R package. A description of the FreeSurfer version and processing pipeline can be found in SI18.

For manuscripts utilizing custom algorithms or software that are central to the research but not yet described in published literature, software must be made available to editors and reviewers. We strongly encourage code deposition in a community repository (e.g. GitHub). See the Nature Portfolio guidelines for submitting code & software for further information.

Data

Policy information about availability of data

All manuscripts must include a data availability statement. This statement should provide the following information, where applicable:

- Accession codes, unique identifiers, or web links for publicly available datasets
- A description of any restrictions on data availability
- For clinical datasets or third party data, please ensure that the statement adheres to our policy

Participant-level data from many datasets are available according to data access policies of the primary studies. Study-level model parameters are available at https://github.com/KaidiK/RESI_BWAS.

Research involving human participants, their data, or biological material

Policy information about studies with human participants or human data. See also policy information about sex, gender (identity/presentation), and sexual orientation and race, ethnicity and racism.

Reporting on sex and gender	We included sex as a biological variable, which was self-reported by study participants.
Reporting on race, ethnicity, or other socially relevant groupings	This study focuses on biological and cognitive associations. Social categories (such as race and ethnicity) were not considered in the analyses.
Population characteristics	We considered age as a population characteristic. In meta-analyses, mean, standard deviation, and kurtosis of age for each sample was included as covariates.
Recruitment	This study used existing data from consortium repositories and did not recruit human subjects.
Ethics oversight	All contributing studies include their own ethical oversight. Because this is secondary research and the data are deidentified when accessed from the primary study repositories it does not constitute human subjects research by NIH policy and does not require ethical approval from the IRB at Vanderbilt University Medical Center.

Note that full information on the approval of the study protocol must also be provided in the manuscript.

Field-specific reporting

Please select the one below that is the best fit for your research. If you are not sure, read the appropriate sections before making your selection.

☐ Life sciences ☒ Behavioural & social sciences ☐ Ecological, evolutionary & environmental sciences

For a reference copy of the document with all sections, see nature.com/documents/nr-reporting-summary-flat.pdf

Behavioural & social sciences study design

All studies must disclose on these points even when the disclosure is negative.

Study description	Meta-analyses based on 63 neuroimaging datasests from Lifespan Brain Chart Consortium (LBCC) were conducted to investigate the influence of study design features that can improve the effect sizes in Brain-wide association studies.
Research sample	Sixty-three neuroimaging datasets, which includes 16 longitudinal datasets and and 46 cross-sectional datasets, from Lifespan Brain Chart Consortium (LBCC) were used in this research.
Sampling strategy	This study used existing data from consortium repositories.
Data collection	This study used existing data from consortium repositories.
Timing	n/a
Data exclusions	The original LBCC dataset includes 123,984 MRI scans from 101,457 human participants across more than 100 studies.. We filtered to the subset of cognitively normal participants whose data were processed using FreeSurfer version 6.1. Studies were curated for the analysis by excluding duplicated observations and studies with less than 4 unique age points, sample size less than 20, and/or only participants of one sex. If there were fewer than three participants having longitudinal observations, only the baseline observations were included and the study was considered cross-sectional. If a subject had changing demographic information during the longitudinal follow-up (e.g., changing biological sex), only the most recent observation was included. We updated the LBCC dataset with the ABCD release 5 resulting in a final dataset that includes 77,695 MRI scans from 60,900 cognitively normal participants who have available total GMV, sGMV and GMV measures across 63 studies, among these, 74,148 MRI scans from 57,538 participants across 43 studies have complete-case regional brain measures.

Non-participation	This study used existing data from consortium repositories and non-participation is reported for those studies at the discretion of the primary study investigators.
Randomization	This is an observational study and no treatment or randomization was applied.

Reporting for specific materials, systems and methods

We require information from authors about some types of materials, experimental systems and methods used in many studies. Here, indicate whether each material, system or method listed is relevant to your study. If you are not sure if a list item applies to your research, read the appropriate section before selecting a response.

Materials & experimental systems

n/a	Involved in the study	
☒	☐	Antibodies
☒	☐	Eukaryotic cell lines
☒	☐	Palaeontology and archaeology
☒	☐	Animals and other organisms
☒	☐	Clinical data
☒	☐	Dual use research of concern
☒	☐	Plants

Methods

n/a	Involved in the study	
☒	☐	ChIP-seq
☒	☐	Flow cytometry
☐	☒	MRI-based neuroimaging

Plants

Seed stocks	n/a
Novel plant genotypes	n/a
Authentication	n/a

Magnetic resonance imaging

Experimental design

Design type	Structural and Functional MRI
Design specifications	n/a
Behavioral performance measures	n/a

Acquisition

Imaging type(s)	Structural and functional
Field strength	Imaging protocol varied across consortia and study sites. See reference below for details.<br>1. Bethlehem R a. I, Seidlitz J, White SR, Vogel JW, Anderson KM, Adamson C, Adler S, Alexopoulos GS, Anagnostou E, Areces-Gonzalez A, Astle DE, Auyeung B, Ayub M, Bae J, Ball G, Baron-Cohen S, Beare R, Bedford SA, Benegal V, Beyer F, Blangero J, Blesa Cábez M, Boardman JP, Borzage M, Bosch-Bayard JF, Bourke N, Calhoun VD, Chakravarty MM, Chen C, Chertavian C, Chetelat G, Chong YS, Cole JH, Corvin A, Costantino M, Courchesne E, Crivello F, Cropley VL, Crosbie J, Crossley N, Delarue M, Delorme R, Desrivieres S, Devenyi GA, Di Biase MA, Dolan R, Donald KA, Donohoe G, Dunlop K, Edwards AD, Elison JT, Ellis CT, Elman JA, Eyler L, Fair DA, Feczko E, Fletcher PC, Fonagy P, Franz CE, Galan-Garcia L, Gholipour A, Giedd J, Gilmore JH, Glahn DC, Goodyer IM, Grant PE, Groenewold NA, Gunning FM, Gur RE, Gur RC, Hammill CF, Hansson O, Hedden T, Heinz A, Henson RN, Heuer K, Hoare J, Holla B, Holmes AJ, Holt R, Huang H, Im K, Ipser J, Jack CR, Jackowski AP, Jia T, Johnson KA, Jones PB, Jones DT, Kahn RS, Karlsson H, Karlsson L, Kawashima R, Kelley EA, Kern S, Kim KW, Kitzbichler MG, Kremen WS, Lalonde F, Landeau B, Lee S, Lerch J, Lewis JD, Li J, Liao W, Liston C, Lombardo MV, Lv J, Lynch C, Mallard TT, Marcelis M, Markello RD, Mathias SR, Mazoyer B, McGuire P, Meaney MJ, Mechelli A, Medic N, Misic B, Morgan SE, Mothersill D, Nigg J, Ong MQW, Ortinau C, Ossenkoppele R, Ouyang M, Palaniyappan L, Paly L, Pan PM, Pantelis C, Park MM, Paus T, Pausova Z, Paz-Linares D, Pichet Binette A, Pierce K, Qian X, Qiu J, Qiu A, Raznahan A, Rittman T, Rodrigue A, Rollins CK, Romero-Garcia R, Ronan L, Rosenberg MD, Rowitch DH, Salum GA, Satterthwaite TD, Schaare HL, Schachar RJ, Schultz AP, Schumann G, Schöll M, Sharp D, Shinohara RT, Skoog I,

Smyser CD, Sperling RA, Stein DJ, Stolicyn A, Suckling J, Sullivan G, Taki Y, Thyreau B, Toro R, Traut N, Tsvetanov KA, Turk-Browne NB, Tuulari JJ, Tzourio C, Vachon-Presseau É, Valdes-Sosa MJ, Valdes-Sosa PA, Valk SL, van Amelsvoort T, Vandekar SN, Vasung L, Victoria LW, Villeneuve S, Villringer A, Vértes PE, Wagstyl K, Wang YS, Warfield SK, Warrier V, Westman E, Westwater ML, Whalley HC, Witte AV, Yang N, Yeo B, Yun H, Zalesky A, Zar HJ, Zettergren A, Zhou JH, Ziauddeen H, Zugman A, Zuo XN, Bullmore ET, Alexander-Bloch AF. Brain charts for the human lifespan. Nature. Nature Publishing Group; 2022 Apr;604(7906):525–533.
2. Feczko E, Conan G, Marek S, Tervo-Clemmens B, Cordova M, Doyle O, Earl E, Perrone A, Sturgeon D, Klein R, Harman G, Kilamovich D, Hermosillo R, Miranda-Dominguez O, Adebimpe A, Bertolero M, Cieslak M, Covitz S, Hendrickson T, Juliano AC, Snider K, Moore LA, Uriartel J, Graham AM, Calabro F, Rosenberg MD, Rapuano KM, Casey BJ, Watts R, Hagler D, Thompson WK, Nichols TE, Hoffman E, Luna B, Garavan H, Satterthwaite TD, Ewing SF, Nagel B, Dosenbach NUF, Fair DA. Adolescent Brain Cognitive Development (ABCD) Community MRI Collection and Utilities [Internet]. bioRxiv; 2021 [cited 2023 Oct 20]. p. 2021.07.09.451638. Available from: https://www.biorxiv.org/content/10.1101/2021.07.09.451638v1

Sequence & imaging parameters: Imaging protocol varied across consortia and study sites. See references above for details.

Area of acquisition: Global and regional brain structural MRI and functional MRI were used

Diffusion MRI: ☐ Used ☒ Not used

Preprocessing

Preprocessing software: Based on Freesurfers recon-all command. We limited our analyses to version 6.1 to reduce software version effects. fMRI data were processed using the abcd-hcp-pipeline version 0.1.3.

Normalization: Based on Freesurfers recon-all command

Normalization template: Based on Freesurfers recon-all command

Noise and artifact removal: Based on Freesurfers recon-all command

Volume censoring: None

Statistical modeling & inference

Model type and settings: We used generalized linear models (GLMs) and generalized estimating equations (GEEs) to estimate study-level effects for cross-sectional and longitudinal studies respectively. We used GLMs to perform meta-analysis of study-level results.

Effect(s) tested: *Define precise effect in terms of the task or stimulus conditions instead of psychological concepts and indicate whether ANOVA or factorial designs were used.*

Specify type of analysis: ☐ Whole brain ☐ ROI-based ☒ Both

Anatomical location(s): Cortical gray matter, subcortical gray matter, white matter, and cortical thickness

Statistic type for inference (See Eklund et al. 2016): We used GLMs and GEEs to model global and regional measures of structure and function. For regional analyses, we applied Benjamini-Hochberg to adjust for multiple comparisons and report effect sizes and replicability curves.

Correction: For regional analyses, we applied the Benjamini-Hochberg procedure to control the False Discovery Rate.

Models & analysis

n/a	Involved in the study	
☒	☐	Functional and/or effective connectivity
☒	☐	Graph analysis
☒	☐	Multivariate modeling or predictive analysis
